# Neuropeptidergic systems in psychiatric disorders

**DOI:** 10.3389/fendo.2025.1654292

**Published:** 2026-01-20

**Authors:** Sadat Hodzic, Therese Riedemann

**Affiliations:** 1 Department of Physiological Genomics, Institute of Physiology, Biomedical Center, Ludwig-Maximilians-Universität München, München, Germany; 2 Center for Physiology, Pathophysiology and Biophysics, Paracelsus Medical University, Salzburg, Austria; 3 Competence Center for Electrophysiology (COMPCEL), Paracelsus Medical University, Salzburg, Austria

**Keywords:** neuropeptides, prefrontal cortex, depression, orexin, oxytocin, somatostatin, vasoactive intestinal peptide, neuropeptide Y

## Abstract

Neuropeptides represent a heterogeneous class of signalling molecules whose release has initially been described in the hypothalamus. Their release often follows a circadian rhythm and basal release may be enhanced by internal and external stressors. Research on the cellular actions of neuropeptides began in the hypothalamus but progressed to the entire brain following observations of neuropeptide and neuropeptide receptor expression throughout the brain. Recent research suggests that the prefrontal cortex (PFC) exhibits particularly high levels of neuropeptides and neuropeptide receptors suggesting that they may modulate cognitive processes necessary for executive function. However, most data on the cellular actions of neuropeptides are derived from non-cortical cells and their relevance to PFC-dependent behaviour is currently not understood. This review aims to bridge the gap between cellular and network actions of neuropeptides and their relevance to behaviour and mood disorders. Therefore, this review summarises the function of the PFC and highlights the effects of selected neuropeptides on cortical processing and PFC-dependent behavioural output. Where available, we compare the actions of neuropeptides in the rodent brain to the human brain and review potential therapeutic benefits of neuropeptides in PFC-dependent neuropsychiatric disorders.

## Introduction

With the great advancement of single-cell and bulk analysis of mRNA transcripts in the brain, neuropeptides have found themselves in the spotlight of recent research (1–4). Recent studies offer a comprehensive and detailed overview of interactions with neuropeptides in different brain regions, in different cell types and even within the same cell promoting our understanding of how neuropeptide systems may modulate brain circuits. Different neuropeptide systems are preferentially expressed in distinct brain areas suggesting a stimulus-specific or circuit-specific mode of action, whose conditions we have not yet fully understood. Neuropeptide and neuropeptide receptor gene expression in the PFC is distinctly higher compared to other cortical or subcortical areas (4), which prompted us to review the possible impact of these neuropeptide systems in the PFC and draw conclusions on how neuropeptide systems may modulate cortical circuits under physiological and pathological conditions. The medial aspect of the PFC (mPFC) is considered the central interface of integrated internal and external stimuli and behavioural responses and lesions to the human mPFC may be associated with symptoms of depressive disorders. The first part of this review is therefore dedicated to the anatomy, connectivity and function of the mPFC in mouse and man. The second part of the review focuses on neuropeptide – neuropeptide receptor systems of the brain and their implications to animal and human physiology and behaviour.

## Anatomical boundaries of the PFC in humans

In humans, the PFC contains Brodman areas (BAs) BA8 to BA14 and BA44-47 (5, 6). These brain areas are characterised by the absence of a granular layer IV and by receiving strong inputs from the mediodorsal (MD) thalamic nucleus. Depending on the literature, the anterior cingulate cortex (ACC) is regarded part of the PFC because (1) it receives input from the MD thalamic nucleus and, (2) with the exception of some aspects of BA32, the ACC does not contain a detectable granular cell layer (7). The ACC in turn is composed of BA24a, BA24b, BA24c, BA25 and surrounded by BA32 and BA33. BA32 is described as “cingulofrontal transition” area, BA33 represents a transition area to more parietal areas (7). The PFC can be divided into a medial and a lateral PFC (lPFC) with BA11l constituting the boundary between the two subdivisions.

## Anatomical boundaries of the PFC in mice and rats

**Figure 1:**
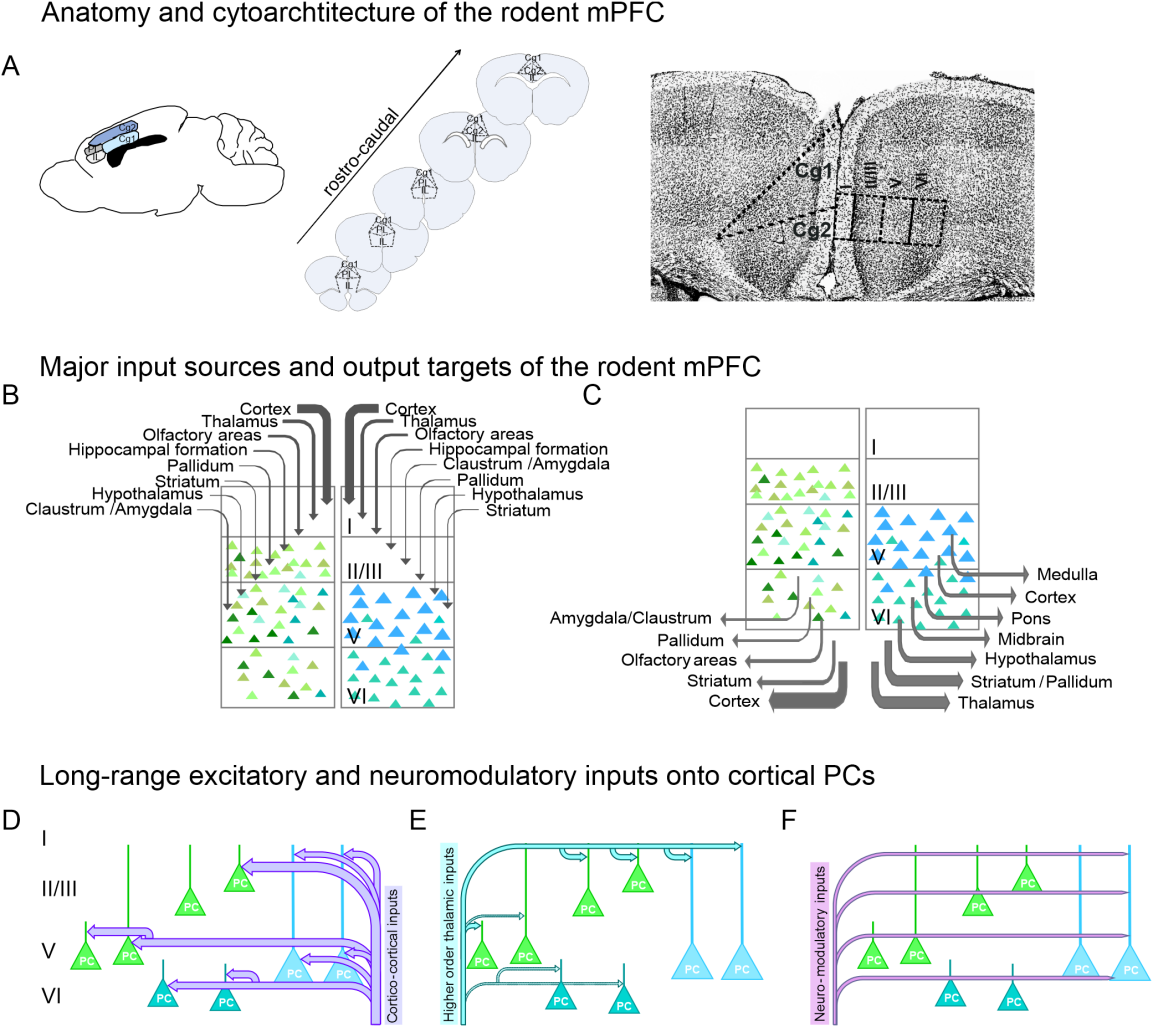
Anatomy and connectivity of the PFC. **(A)** Schematised drawing of the anatomy and rostro-caudal extent of the rodent mPFC (left and middle panel). Confocal image of the ACC showing the dorsal (Cg1) and ventral (Cg2) subdivision. Nuclei were stained for the neuronal marker NeuN. Cortical layers I-VI indicated by Roman numbers. **(B)** Scheme showing the major input sources (left) and output targets (right) of the rodent mPFC. Strength of connectivity indicated by arrow thickness. Roman numbers indicate cortical layers. **(C)** Cartoon showing layer-specific cortico-cortical inputs onto PCs. Input strength represented by arrow thickness. **(D, E)** Schematised drawing with cortico-cortical **(D)** and higher order thalamo-cortical **(E)** inputs onto cortical PCs. Input strength represented by arrow thickness. Roman numbers indicate cortical layers. **(F)** Overview of neuro-modulatory inputs onto cortical PCs.

The brain areas of the rodent PFC are less clearly outlined (8–12). According to Van de Werd (10), the mouse PFC contains the following brain areas: (1) Frontal area 2, (2) the prelimbic (PL) and (3) infralimbic (IL) cortex, (4) the dorsal (Cg1) and ventral (Cg2) aspect of the cingulate area, (5) the medial orbital area, (6) the dysgranular insular areas, (7) the dorsal and ventral aspect of the dorsal agranular area, (8) the posterior agranular insular area, (9) the lateral and medial orbital area, (10) the ventrolateral orbital and (11) the ventral orbital area. Although there exist slight cytological and anatomical differences (13), similar anatomical boundaries are described for the rat PFC (12). The medial aspects of the PFC include the Frontal area 2, the PL and IL cortex, Cg1, Cg2, and the ventral and medial orbital area. However, in most atlases, the ventral orbital area is not recognised as a separate area and the medial orbital area is often included in the PL (11), which is why Cg1 and Cg2, the PL and IL cortex are commonly regarded as the mouse/rat mPFC (**Figure 1A**).

## Connectivity of the mPFC

### Long-range inputs from other brain areas

The mPFC receives strong afferent inputs mainly from other parts of the cortex, especially from the agranular orbital, the secondary motor and the retrosplenial cortex (14–19) (**Figures 1B, D**). Projections from sensory cortex areas to the mPFC are strongest from the somatosensory cortex, while inputs from the visual and auditory cortex are rather weak (8, 14, 17, 18, 20). As mentioned before, the mPFC receives robust inputs from the MD thalamic nucleus, followed by inputs from the ventromedial (VM) and anteromedial (AM) thalamic nucleus and the ventral anterior-lateral complex of the thalamus (14, 21–25). These thalamic inputs preferentially innervate the distal apical dendrites of prefrontal pyramidal cells (PCs) (**Figure 1E**). These connections between adjacent frontal cortex (FC) areas and the mPFC as well as between the mPFC and thalamic nuclei are part of the so-called central executive network that allows performance monitoring and top-down attentional control.

In addition, the mPFC receives inputs from the hippocampus, basal ganglia and the amygdala (14, 24, 26, 27). Further viral tracing and functional studies show that the mPFC receives neuromodulatory input from the hypothalamus, the basal forebrain and from brain stem nuclei (e.g. nucleus raphe, locus coeruleus (LC)) (14, 28–30) to enable context- and state-dependent modulations of ongoing tasks (**Figure 1F**).

### mPFC target areas

The mPFC exhibits dense efferent projections to cortical and subcortical brain areas highlighting its central role in executive function (**Figure 1C**). The major output targets of the mPFC are adjacent (pre-)frontal cortex areas, the retrosplenial cortex, the secondary visual cortex (V2), the parietal associative cortex, and the ectorhinal and perirhinal cortex. mPFC-thalamic projections virtually reach all thalamic nuclei, especially the AM thalamic nucleus, the reticular thalamic nucleus, the VM and MD thalamic nuclei and the dorsal part of the zona incerta. Major subcortical target structures of the mPFC include the striatum, the nucleus accumbens, the claustrum, the amygdala, the septum, the hypothalamus, the periaqueductal gray (PAG), the superior colliculus, the pons, the tectum and the nucleus raphe (14, 19, 31, 32).

## PFC cytoarchitecture

**Figure 2:**
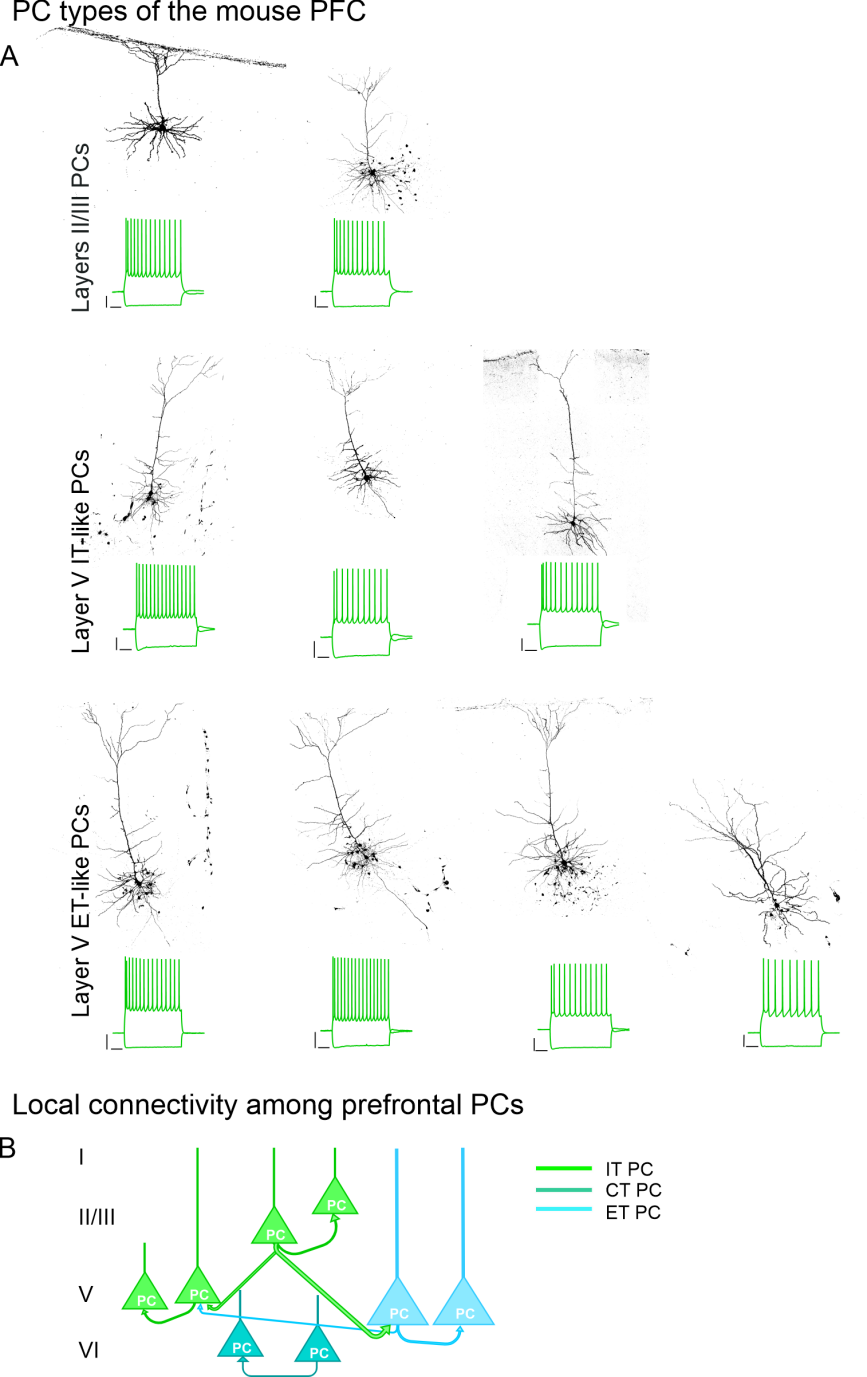
Overview of morphological/electrophysiological PC types in the mouse PFC. **(A)** Exemplar illustrations of LII/III PCs (upper panel), LV intratelencephalic-like (IT-like) PCs (middle panel) and LV extratelencephalic-like (ET-like) PCs (lower panel) with corresponding voltage recordings in response to a hyperpolarising and depolarising current step, respectively. Scalebar: 20 mV/200 ms. **(B)** Schematised overview of local connectivity among prefrontal PCs. Strength of synaptic coupling indicated by thickness of arrow. IT PC, Intratelencephalic PC; CT, Cortico-thalamic PC; ET PC, Extratelencephalic PC. Roman numbers indicate cortical layers.

As mentioned above, the mPFC is characterised by the absence of a cortical layer IV (LIV). In contrast, LV is expanded compared to primary sensory brain areas and harbours a small number of large PCs projecting to the spinal cord (7). Cytological studies revealed that the soma size of neurons from the rostral parts is larger compared to the more caudal aspects of the mPFC and immunoreactivity to acetylcholinesterase is larger in the ACC compared to the midcingulate cortex (MCC) (7). Around 80% of neurons within the mPFC are excitatory projection neurons whereas local interneurons (INs) represent up to 20% of all neurons (33–35). PCs are found in LII-VI, local INs are found in all cortical layers. Either neuron class is sub-divided into functionally distinct subtypes of neurons. PCs are further classified according to their projection targets that lie inside or outside the telencephalon. PCs whose projections mainly reach the telencephalon are named intra-telencephalic (IT) PCs. PCs whose projection targets lie outside the telecenphalon (i.e. extratelencephalic (ET) PCs) are further subdivided into PCs that mainly project to the brain stem and spinal cord (pyramidal tract (PT) PCs) and PCs with major collaterals to the thalamus or striatum. Using retrograde tracers and virus-injections in mice, it could be shown that PCs not only have distinct projection targets, most of them also have a layer-specific localisation within the mPFC (26, 36): (1) IT PCs that project to the basolateral nucleus of the amygdala (BLA) are mainly found in LII. (2) IT PCs that project to the ipsilateral striatum are preferentially found in LII, LIII and LVa. (3) IT PCs projecting to the contralateral striatum are fewer in numbers and are concentrated in LVa. (4) PCs projecting to the contralateral PFC are majorly found in LII-V, whereas (5) PCs projecting to the claustrum are fewer in numbers and evenly distributed across LIII-VI. In contrast, (6) ET PCs that project to brain stem nuclei are restricted to the dorsal part of LVb, whereas (7) ET PCs with major collaterals to the thalamus are concentrated in LVI. A multi-modal analysis of different PC types suggests distinct transcriptomic, morphological and electrophysiological profiles (37). Supragranular IT PCs tend to be regular spiking, tend to have a wide apical tuft, exhibit a more hyperpolarised membrane potential and tend to have a smaller I_f_ conductance due to a smaller hyperpolarisation-activated cyclic nucleotide gated (HCN) channel activity. In contrast, LV IT PCs tend to have a narrow apical dendrite that may or may not extend to LI and tend to have a more depolarised membrane potential and a larger I_f_ conductance. LV/LVI ET cells in turn tend to have a higher instantaneous action potential frequency, a larger soma diameter and a thick-tufted apical dendrite that may or may not extend to LI (38–43) (**Figure 2A**).

## Local mPFC connectivity

### Excitatory connections

PCs of the mPFC also connect to neighbouring PCs within their home cortex. Local connections between neighbouring PCs are altogether scarce. The average synaptic coupling probability is around 10%, however, there are slight differences in connectivity (**Figure 2B**): (1) LII/III IT PCs project preferentially to LV ET or IT PCs. (2) Synaptic coupling rates within the same cortical layer tend to be lower compared to across layer coupling rates (44, 45).

## Inhibitory cell types and their excitatory and inhibitory connectivity

**Figure 3:**
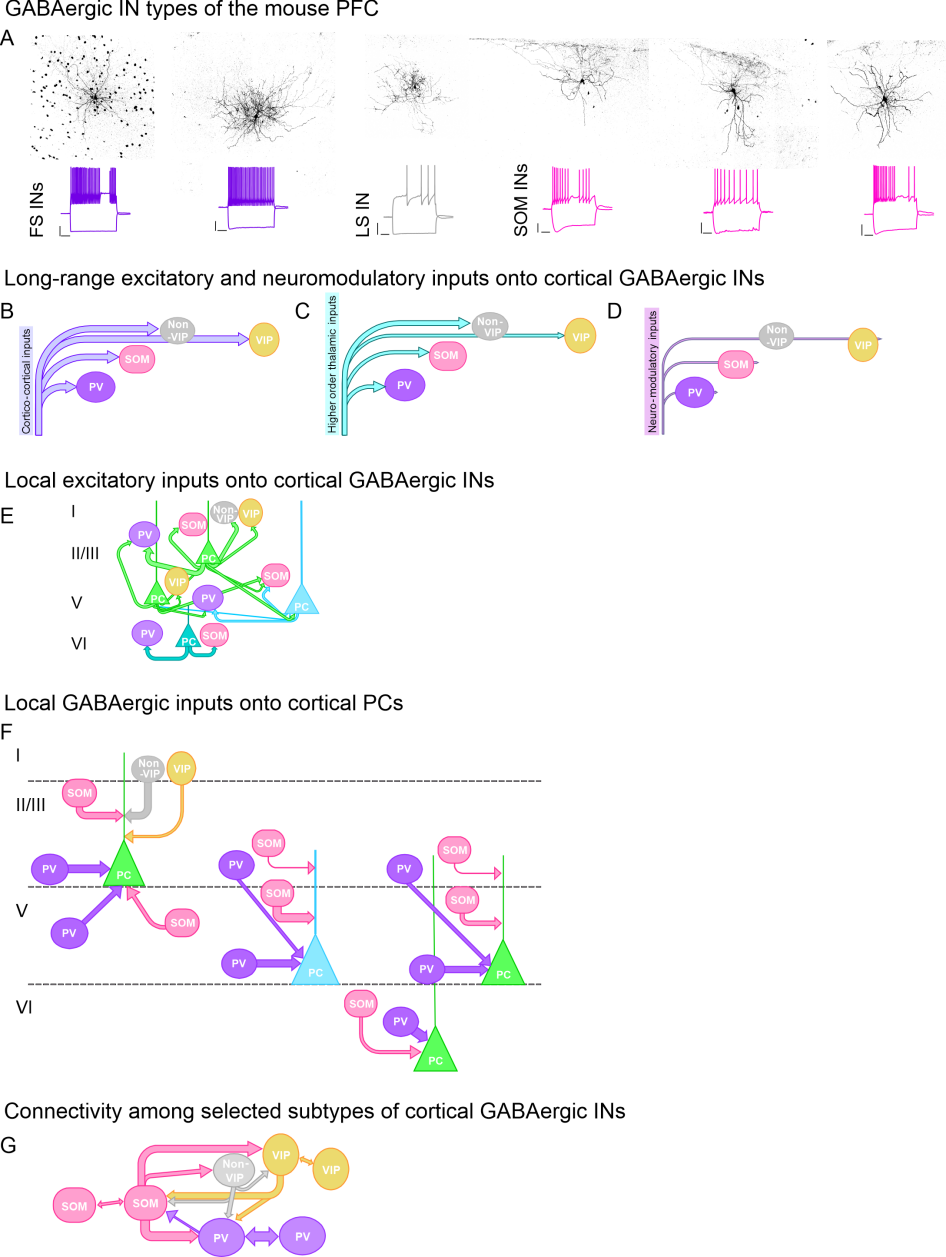
Overview of GABAergic interneurons (INs) and their connectivity in the PFC. **(A)** Exemplar illustrations of different IN types (as indicated) of the mouse PFC with corresponding voltage recordings in response to a hyperpolarising and depolarising current step, respectively. Scalebar: 20 mV/200 ms. FS: fast-spiking, LS: Late-spiking. **(B)** Cartoon showing layer-specific cortico-cortical inputs onto GABAergic INs. Input strength represented by arrow thickness. **(C)** Schematised drawing with inputs from higher order thalamic nuclei onto GABAergic INs. Input strength represented by arrow thickness. **(D)** Overview of neuro-modulatory inputs onto GABAergic INs. **(E)** Schematised overview of local connectivity between cortical PCs and different types of GABAergic INs. Green: Intratelencephalic PC, blue: Extratelencephalic PC, cyan: Cortico-thalamic PC. Strength of synaptic coupling indicated by thickness of arrows. Roman numbers indicate cortical layers. **(F)** Schematised overview of different GABAergic inputs onto cortical PCs. Strength of synaptic coupling indicated by thickness of arrows. Green: Intratelencephalic PC, blue: Extratelencephalic PC. Roman numbers indicate cortical layers. **(G)** Cartoon showing local connectivity among different types of GABAergic INs. Strength of synaptic coupling indicated by arrow thickness. Electrical coupling indicated by double-headed arrow.

Classification studies of the last 70 years have greatly helped to find organising principles of GABAergic INs. Mostly on the basis of biochemical and transcriptomic profiles, electrophysiological properties, morphological characteristics and short-term synaptic plasticity rules, GABAergic INs are have been grouped into 4 overarching classes: INs expressing (1) parvalbumin (PV) or (2) somatostatin (SOM) or INs expressing (3) vasoactive intestinal peptide (VIP) or (4) not. The latter group (non-VIP-expressing INs) can be further split into INs that either express reelin or Id2 (34, 46–49). All IN classes are found in the mPFC (**Figure 3A**). Each IN class receives long-range excitatory inputs from other cortex areas, however VIP INs tend to receive slightly stronger cortico-cortical inputs (17, 18, 50). In addition, all IN types receive higher order thalamic input, that is on average more pronounced in PV-INs and non-VIP-INs compared to SOM-INs and VIP-INs (17, 18, 51). In addition, the activity of all IN types is modulated by cholinergic, monoaminergic or peptidergic inputs (52–60) (**Figures 3B–D**).

Local excitatory inputs onto GABAergic INs are provided by neighbouring PCs. Compared to PC-PC connectivity, synaptic coupling between GABAergic INs and PCs is a lot higher (**Figure 3E**). The synaptic coupling ratio between supragranular PCs and PV-INs is around 40%, and that between supragranular PCs and SOM-INs is roughly 20-30%. VIP-INs tend to receive slightly fewer synaptic inputs from neighbouring PCs, roughly 15%. In addition, translaminar connectivity is rare but may occur between infragranular PCs and supragranular PV-INs or between supragranular PCs and infragranular SOM-INs. Infragranular connectivity between PCs and INs is highest between LVI PCs and LVI SOM-INs or PV-INs (44, 45).

GABAergic inhibition of PCs in turn is mostly provided by PV-INs, SOM-INs and non-VIP INs, whereas VIP-INs tend to preferentially inhibit other IN types, particularly SOM-INs and PV-INs (**Figure 3F**). PV-INs exhibit a high coupling ratio and exert powerful somatic inhibition of PCs. SOM-INs and non VIP-INs have a slightly lower synaptic connectivity with PCs and preferentially target the distal dendrites of postsynaptic PCs. SOM-INs further inhibit all other GABAergic INs whereas PV-INs do not provide major inhibition of other INs. All IN types are further connected via electrical coupling to differing degrees (44) (**Figure 3G**).

Collectively, mPFC connectivity suggests, that integrated inputs from the thalamus and from adjacent brain areas converge onto mPFC PCs. In a feedforward inhibitory motif, these long-range inputs are modulated by PV-INs, non-VIP INs and SOM-INs. In addition, SOM-INs provide feedback inhibition of neighbouring PCs (61) whereas VIP-INs are primarily in charge of PC disinhibition via their preferential inhibition of SOM-INs (62, 63). This long-range connectivity between the mPFC and other cortex areas lays the ground for a comparison and integration of different stimuli and represents a feedback loop for monitoring behavioural output. The local GABAergic network within the mPFC in turn is the basis of precisely-timed spike occurrence allowing synchronised activity across multiple neurons to enable execution of mPFC-dependent behavioural outputs (64, 65).

## The PFC and goal-directed behaviour

The mPFC is considered the central interface of integrated internal and external stimuli and behavioural output. On the basis of present stimuli and past experiences, the PFC predicts the consequences of each behavioural response and must therefore be capable of behavioural flexibility, i.e. the PFC possesses the ability of situational cognitive capture in combination with executive attention to compute a behavioural response and its future implications to self and others (66–68). The nature of PFC-mediated behavioural output is therefore goal-directed and aimed at optimising outcomes.

## Behaviour-related neurophysiological activity of the PFC

The PFC-dependent optimisation of future actions by evaluating current behavioural outputs relies on feedback information. Electroencephalography (EEG) recordings provided evidence for the existence of a negative brain potential in the mPFC in response to external feedback signalling either a negative and/or unfavourable consequence of one’s behavioural output or in response to a participant’s subjective feeling of having performed incorrectly (66, 69, 70). A temporally similar negative potential, the error-related negativity (ERN), is observed in response-locked, event-related potentials (ERPs) in participants solving a choice reaction time task incorrectly (71–81). The ERN is a tripolar potential with an initial negative peak, followed by a positive and a subsequent negative potential. The time intervals between positive and negative peaks suggest that it may represent error-locked theta band (4-8 Hz) oscillatory activity. Increases in theta band power could indeed be observed immediately after the occurrence of an error (82–84), followed by a slowing down of reaction times due to a selective suppression of corticospinal excitability immediately after the occurrence of an error and during the preparation of the next action (85). These findings corroborate the idea that the ERN is part of a feedback loop activity that constantly monitors performance and adjusts behavioural outputs (69, 86, 87). It occurs independently of sensory feedback (88, 89) and of the mode of behavioural output (e.g. manual versus vocal) (90–92).

Direct recordings of single neurons in the human PFC provide evidence for the existence of (1) conflict neurons whose activity pattern is correlated with interference presentation, (2) error neurons that signal endogenous errors before the presentation of external feedback and without the presentation of an additional sensory signal and (3) error-integrating neurons whose activity upon current stimulus presentation is a consequence of the preceding conflicting stimulus (93, 94). By combining single-neuron recordings with intracranial EEG recordings, it was further shown that the ERN amplitude correlates with the spiking rate of error neurons, most of which have been identified as PCs based on waveform analysis (93).

Functional brain imaging studies identified the dorsal ACC as a key neural substrate for this feedback loop (94–99). This finding is supported by behavioural symptoms associated with unilateral or bilateral lesions of the human cingulate gyrus: Lesions to the dorsomedial part of the PFC, especially the ACC, lead to failures in error monitoring but also to emotional dysbalance, inattention, autonomic dysregulation, reduced response production, and, if extreme, to abulia or akinetic mutism (**Table 1**). In contrast, damage to the ventromedial part of the PFC results in impaired affect regulation, motivation and decision-making (20, 100–102). which may lead to a higher risk of developing a major depressive disorder (MDD) (103, 104).

**Table 1:** Correlation of PFC lesions with behavioural symptoms in humans.

Brain region	Lesion type/ Disorder	Behavioural symptoms/Outcome
Bilateral gyrus cinguli	Tumor infiltration to both gyrus cinguli and midline regions	Akinetic mutism (747)
Right ACC and left CC	Left anterior artery occlusion and stenosis of right anterior artery	Akinetic mutism (747)
Bilateral ACC and fornix	Bilateral infarction of ACC	Lack of concentration Impulsivity Emotional indifference (748)
Bilateral ACC, left F1 premotor and bilateral medial orbital cortex	Occlusion of ascending branches of both anterior cerebral arteries	Akinetic mutism Monotonous speech Lack of concentration Compulsory behavior (749)
CC	Cingulotomy due to neoplastic and chronic pain	Improvements in pain severity (750–760) Reduced self-initiated speech (750, 751) Impaired error detection on Stroop interference paradigm (186, 750) Reduced attention span (750) Deficits of focused and sustained attention (751) No impairment of IQ (750) No impairment of overall language performance from the Boston Aphasia Battery (750) No motor deficits (750)
ACC-MCC	Epilepsy Implantation of intracranial electrodes in two human patients and subsequent analysis of symptoms following ACC stimulation	Appearance of autonomic symptoms (shakiness and hot flashes) in the upper chest region Increase of heart rate Feelings of perseverance (761)
Bilateral ACC	Cingulotomy due to chronic depression or OCD	Impaired error detection on Stroop interference paradigm (762–764) Reduced verbal fluency (762) Reduced emotion recognition accuracy (762, 763) IQ unaffected (765) Improved spatial working memory following 12 months of cingulotomy (765) Impaired generation of novel sequences and mental operations (764) Relieve of OCD symptoms (764) Relieve of depressive symptoms (763, 764)
Right dorsal ACC	Cingulotomy due to OCD	Reduced OCD symptoms (766)
Right cingulate BA24	Stroke/Trauma/Tumor	Prolonged simple reaction times (767)
Right cingulate BA23, BA9, bA46	Stroke/Trauma/Tumor	Prolonged choice and prepare reaction times (768)
PFC	Focal prefrontal lesion	Increased reaction times (768)
ACC	Focal ACC damage due to tumor resection (right)	Normal Stroop interference (vocal output not manual output) (769)
ACC	Focal damage to left rostral-mid ACC due to (probably) occlusion of pericallosal branch of anterior cerebral artery	Reduced Stroop performance via spoken responses (not manual responses) (769)
ACG	Traumatic brain injury in veterans	Impaired recognition of pleasant emotions (770)

ACC, anterior cingulate cortex; AC, anterior cingulate gyrus; BA, Brodmann area; CC, cingulate cortex; PFC, prefrontal cortex; OCD, obsessive-compulsive disorder.

## Relevance of mPFC to MDD

MDD is characterised by affective, cognitive and autonomic dysfunction. These present as, among others, pervasive feelings of hopelessness, diminished interest in pleasurable activities, fatigue, anhedonia and bias towards negative stimuli.

The depressive cognitive triad is a possible reason for MDD and describes the following symptoms: (1) negative beliefs and judgments about oneself; (2) tendency to interpret current experiences in a negative way; (3) negative judgments and predictions about the future (105–107).

Interestingly, depressed and non-depressed people do not differ significantly in their initial responses to negative life events, however, they differ in their ability to cope with these events, i.e. in their resilience towards stressors (108, 109). Attention bias may result from the difficulty of disengaging attention away from aversive stimuli, which is associated with reduced activation in the upper parietal lobe, the ventrolateral (vlPFC) and the dorsolateral PFC (dlPFC). The bias in emotional processing is associated with a particular amygdala reactivity, left dlPFC hypoactivity and right dlPFC hyperactivity. In turn, the inhibition of negative information may be disturbed due to the abnormal ACC activity. Reduced ability to experience positive affect and lower sensitivity to rewards are associated with decreased activity of the nucleus accumbens and the PFC. Thinking bias and a tendency to ruminate are associated with hyperactivity of the functional network including the amygdala, hippocampus and PFC, offering an explanation of why deep brain stimulation, including stimulation of the subgenual cingulate cortex may be helpful in reducing depressive symptoms and treating drug-resistant depression (110).

EEG studies have shown, that, *inter alia*, symptoms of depression are associated with a reduced amplitude of the positive peak of the ERN suggesting an impaired capacity for error-monitoring and subsequent behavioural adjustments in depressed patients (111–113).

Altogether, impaired mPFC signalling represents one of many factors contributing to the pathogenesis of MDD: Nonetheless, dysfunctions of the mPFC may set the course for developing MDD and other neuropsychiatric diseases.

Studies have shown that rodents may exhibit symptoms of depressive-like disorders and different experimental paradigms allow a quantitative assessment of potentially depressive behaviours (114–124). As in humans, depressive-like symptoms in rodents may be induced by exposure to chronic unpredictable stress (CUS) that consolidates an experience of learned helplessness and despair in the animal. Alternatively, depressive-like symptoms can be observed in different genetic mouse models or after, among others, surgical, pharmacological or social manipulations. An overview of behavioural tests whereby symptoms of despair and anhedonia are determined in the rodent animal is provided in **Table 2**.

**Table 2:** Animal tests for depressive-like and/or anxiety-like behaviours in rodents.

Behavioural test	Test design	Interpretation
Sucrose preference test	Animals are given a choice of plain drinking water or sucrose enriched water (usual sucrose concentration: 0.25 – 2%). The sucrose preference is usually indicated as ratio of consumed sucrose water volume over the total volume of liquid consumed.	Reduced sucrose preference as indicator of anhedonia
Learned helplessness	During the training, rodents are exposed to an inescapable and stressful situation (e.g. uncontrollable foot shock). Two groups of control animals are either not exposed to a stressful situation or exposed to a stressful situation where they can escape from. During the test, both stress-exposed groups can escape from the stressful situation and behavioural readouts include: Number of attempts to escape the stressful situation and latency to escape.	Increased latency to escape and/or fewer attempts to escape the stressful situation suggest increased levels of despair
Tail suspension test	Rodents are suspended by their tail for several minutes. Usual behavioural readouts are indicated as units of time and include: Immobility time, latency to immobility, swinging and/or curling time.	Increased latency to immobility and/or decreased duration to fight the stressful situation are considered as increased signs of despair
Forced swim test	Rodents are forced to swim and keep their head above the water level by placing them in a water-filled glass cylinder for several minutes. Usual behavioural readouts are indicated as units of time and include: Swimming, struggling and immobility	Fewer attempts of trying to escape the stressful situation are regarded as higher levels of despair
Sucrose splash test	Rodents are sprayed with sucrose-containing (usually 10%) water to induce grooming behaviour. The behavioural endpoint is usually given in units of time as duration of grooming.	Reduced evoked (and/or spontaneous) grooming behaviour regarded as heightened level of apathy
Social interaction test	Animals are presented a novel conspecific in an open-field arena that they have previously investigated. Social interaction is usually quantified as ratio of time spent in area with the conspecific and without it.	Less interest in novel conspecific suggests decreased level of sociability
Crawley’s sociability test	Modified social interaction test. The test animal is free to choose an interaction with a novel conspecific or with a familiar one. Social interaction is quantified as ratio of time spent with the novel conspecific and with familiar conspecific and the total time spent with novel conspecific.	Less interest in novel conspecific suggests decreased level of sociability
Open field test	Animals are placed in an open field maze that consists of a brightly-lit wall-enclosed area of sufficient size. Typical behavioural readouts are: Locomotor activity (total distance travelled, track path, time spent in the outer vs. inner zone of the area) and amount of defecation quantified as number of fecal boli.	Increased time spent in the outer zone of the open field maze and increased defecation as signs of anxiety
Elevated plus maze test	Animals are placed at the intersection of an elevated plus sign-shaped maze consisting of two open and two closed arms. Behavioural parameters include: Time spent in the open versus the closed arms, entries made to the open versus the closed arms, amount of defecation, freezing time, number of rears, and number/duration of stretched attend posture	Increased time spent in the closed arms indicates higher levels of anxiety
Light-dark box	Animals are placed in a maze consisting of two chambers of different sizes connected by an opening door: 1) A smaller (usually 1/3 of total area) is dark and 2) a larger chamber (usually 2/3 of total area) is brightly lit. Mice are usually placed in the center of the well illuminated chamber and test typically lasts 5 minutes. Behavioural endpoints include: Latency to enter dark chamber, number of transitions, total time spent in light/dark or ratio thereof and number of rears.	Increased time spent in the dark chamber of the light-dark box suggestive of heightened anxiety

Given that neuropeptide and neuropeptide receptor gene expression in the PFC is distinctly higher compared to other cortical or subcortical areas (4), neuropeptide-neuropeptide receptor dysfunction/dysbalance may contribute to the development of mood disorders.

We will therefore first provide some background information on neuropeptides and second review the physiological actions of selected neuropeptides in the (mostly) rodent brain and, where possible, compare these to effects in humans.

## Hormones and neuropeptides

The term hormone was coined by Ernest Starling at the beginning of the 20^th^ century and defines substances that are transported via the blood to have a specific effect on target organs or cells. He and William Bayliss hypothesised the presence of a factor released by mucosal cells of the duodenum that induces the secretion of digestive enzymes from the denervated pancreas in response to gastric acid secretion. The substance was hence called secretin. Around 20 years later, Andrew Conway Ivy and Eric Oldberg found a substance that induced gall bladder contractions and they named it cholecystokinin (CCK). Again 40 years later, the amino acid sequence of CCK was identified by Viktor Mutt and Erik Jorpes (125).

We now know that most, if not all, gastro-intestinal (GI) hormones and peptides along with their cognate receptors are also expressed within the central nervous system (CNS), where they majorly act locally on postsynaptic target cells. In addition, peptides associated with energy homeostasis such as leptin from adipose tissue, ghrelin from the stomach, and insulin from the pancreas are not only synthesised in the brain but have been shown to act on selective receptors within the CNS to control food intake and metabolism.

Neuropeptides are defined as peptide transmitters that are released by neurons, often in combination with a ‘classic’ neurotransmitter (126, 127). Unlike classic neurotransmitters however, neuropeptides appear to be released only in response to a high frequency discharge of presynaptic action potentials (APs) (128–131). Basal neuropeptide release underlies, in many cases, a circadian rhythm (132–134). Hormonal changes (e.g. those associated with pregnancy or puberty), external or internal stressors (135–151) or sensory stimuli may further promote context-dependent release (152–156). On a cellular level, the following stimuli may influence the release of neuropeptides:

1. Intracellular Ca^2+^ levels (157–159).
2. Membrane depolarisation and/or activation of glutamatergic receptors (160–167).
3. Other neurotransmitters such as dopamine, norepinephrine, acetylcholine or serotonin (168–172)
4. Presynaptic GABA_B_ receptors (173–178).
5. Auto-inhibition or auto-promotion as well as cross-inhibition or cross-promotion (179–192).
6. Internal and/or external stressors (135, 136, 138, 139, 141, 142, 144–151).

Once released from presynaptic sites, neuropeptides tend to bind with high specificity to the extracellular loop binding pocket of their cognate GPCR (193).

**Figure 4:**
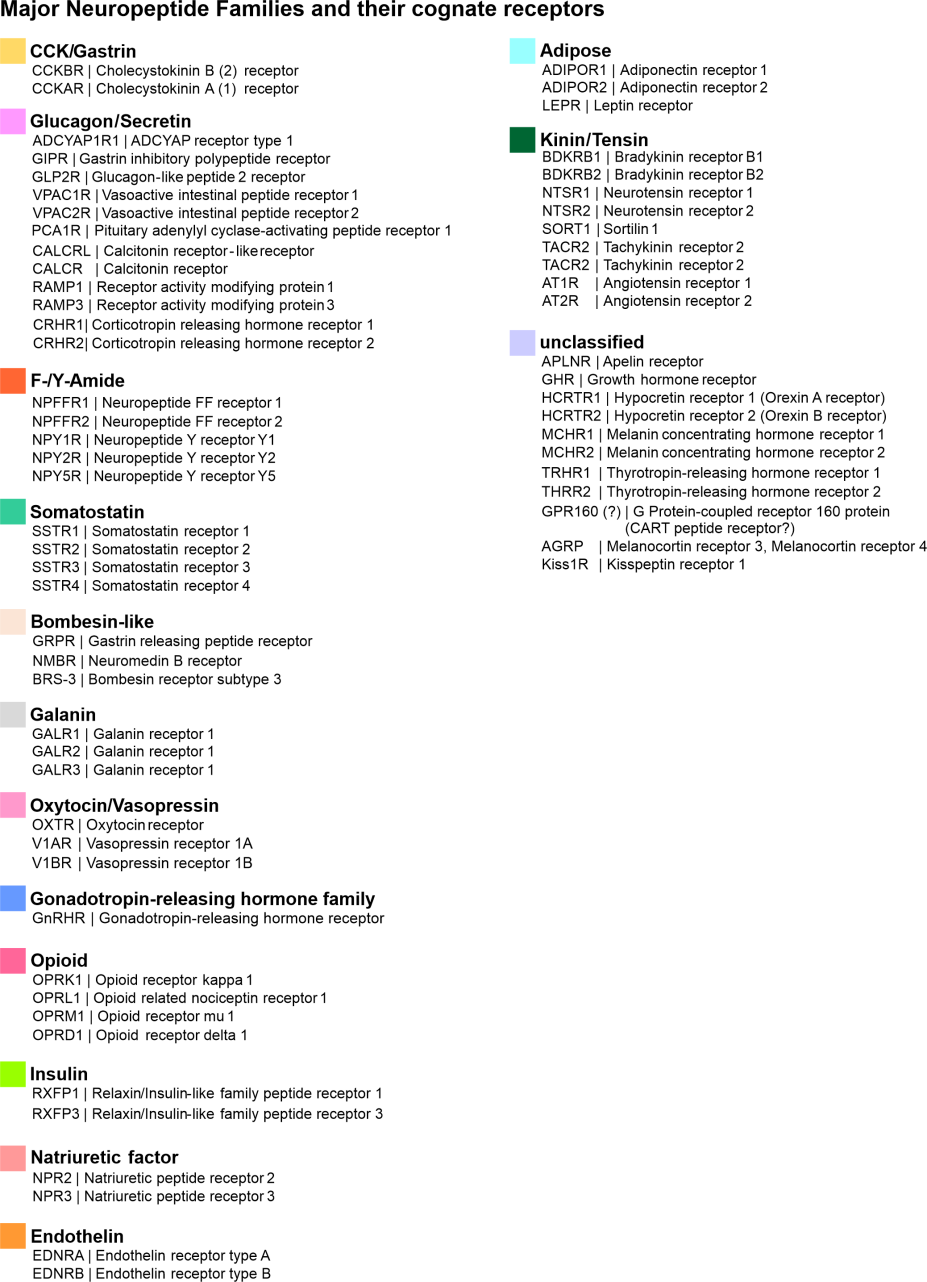
Overview of major neuropeptide families and their cognate receptors.

**Figure 5:**
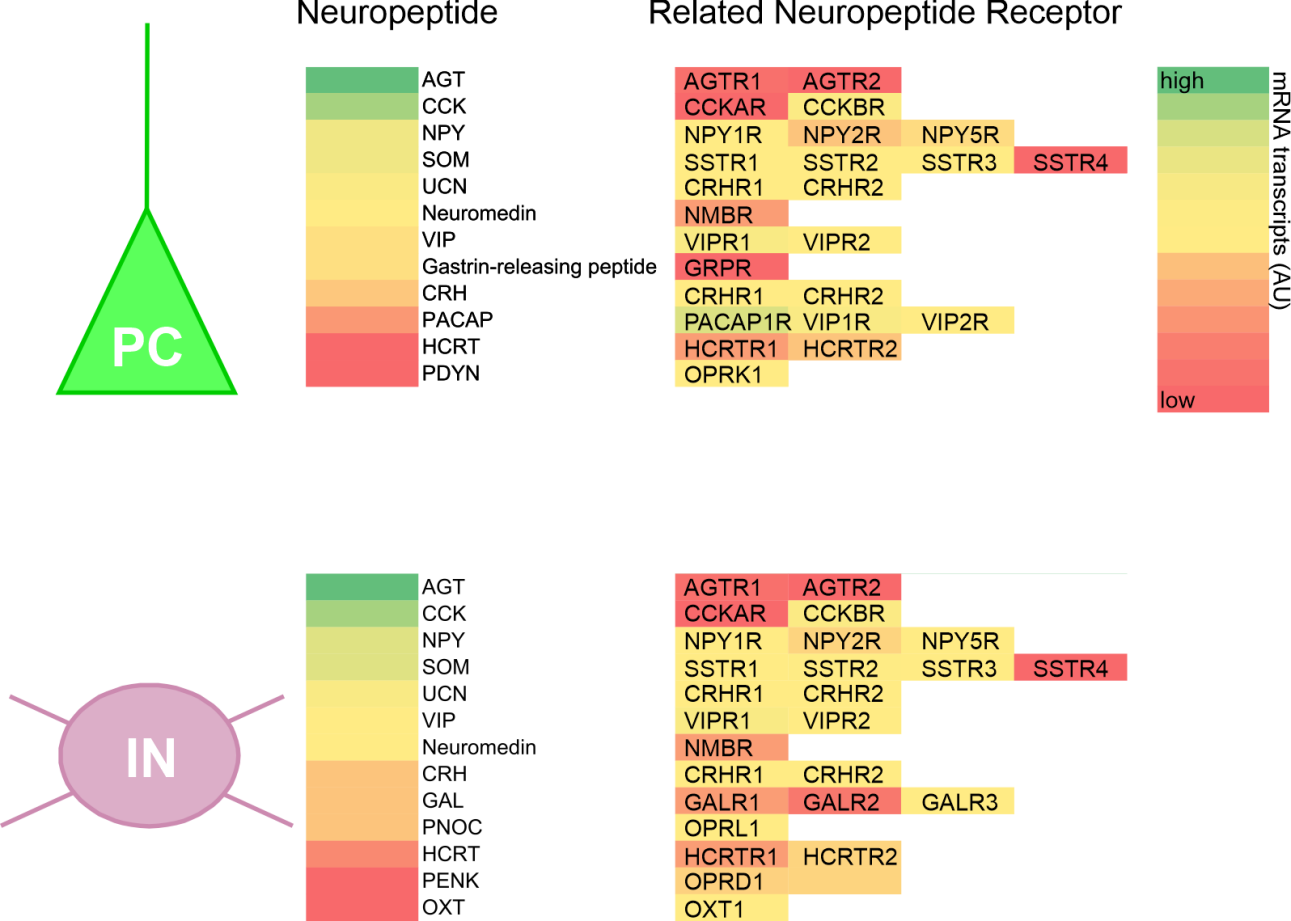
Neuropeptide/neuropeptide receptor expression in prefrontal PCs and GABAergic INs. Schematised drawing showing neuropeptide/neuropeptide receptor mRNA levels in cortical PCs (upper panel) and GABAergic INs (lower panel) based on single-cell transcriptomic data. AGT, Angiotensinogen; AGTR1, Angiotensinogen receptor 1; CCK, cholecystokinin; CRH, corticotropin-releasing hormone; CRHR, corticotropin-releasing hormone receptor; GAL, Galanin; HCRT, hypocretin/orexin; NPY, neuropeptide Y; OXT, oxytocin; PACAP, pituitary adenylyl cyclase-activating peptide; PDYN, prodynorphin; PENK, proenkephalin; PNOC, prepronociceptin; SOM, somatostatin; UCN, urocortin; VIP, vasoactive intestinal peptide.

The class of neuropeptides is a diverse one and comprises around 100 different peptides (194). In mammals, neuropeptides can be classified into around 15 distinct neuropeptide families on the basis of structural and/or functional similarities (**Figure 4**). The great majority of neuropeptides mediate their actions via their cognate G Protein-coupled receptor (GPCR), hence, neuropeptidergic modulation of neuronal activity occurs on longer timescales and is suggested to influence a greater number of target neurons. **Figure 5** schematises selected neuropeptide and neuropeptide receptor expression in PCs or GABAergic INs of the human PFC.

Importantly, exposure to identical stimuli under experimental conditions leads to the release of different types of neuropeptides. Therefore, crosstalk between different neuropeptide systems is likely to occur on neuropeptide-releasing and on ‘neuropeptide-receiving’ target cells, making the effects of neuropeptides highly complex.

The many functions of neuropeptides/neuropeptide receptors described in the following sections provide an idea of their multi-level actions on information processing in the brain. However, despite a wealth of literature on the cellular actions of neuropeptides on neurons, it is still unclear how neuropeptides or neuropeptide receptors may modulate physiological and pathophysiological mPFC-dependent behavioural output. In light of the evidence that mPFC GABAergic INs and PCs coexpress multiple neuropeptides and neuropeptide receptors, a better understanding of these neuropeptide systems in the mPFC is needed for future pharmacological interventions of neuropsychiatric disorders.

## CCK

### Discovery and expression in the brain

To date, 5 different bioactive CCK peptides have been identified (CCK-5, CCK-8, CCK-22, CCK-33, CCK-58, CCK-83). With the exception of CCK-5, these peptides occur either in a sulphated or non-sulphated form (195). Immunoreactivity against CCK-like peptides in the brain was initially described in 1976 (196). Further studies have reported the expression of CCK peptides in PCs and GABAergic INs (197–203) alike making CCK (CCK-8 and CCK-5) one of the most abundant neuropeptides of the human and rodent neocortex (2, 4, 204–209). CCK and gastrin belong to the same family of peptides based on their identical C-terminal amino-acid sequence (205, 210, 211). Both peptides mediate their actions via two different CCK receptors: The CCKA or CCK1 receptor (CCKAR, CCK1R) binds amidated and sulphated CCK peptides with high affinity but no gastrins. It is mostly expressed in the myenteric plexus, anterior pituitary and midbrain. CCKBR or CCK2R is expressed in the stomach, pancreas and to a high degree in the brain (2, 4, 212–214) and binds to sulphated and non-sulphated CCKs and also to gastrins (205).

### Postsynaptic actions of CCK

Initial evidence for CCK-responsive cortical neurons was provided in 1979 showing that iontophoretically applied CCK-8 leads to a robust increase in spontaneous AP discharge in the great majority of recorded cells (215). These results have meanwhile been confirmed by others in PCs and GABAergic INs of different brain regions and provided evidence for enhanced glutamatergic and GABAergic neurotransmission as a result of CCK-induced increases in excitability (203, 216–225).

By combining electrophysiological recordings with pharmacology, immunochemistry and use of CCKB knockout (KO) mice, it was further shown that this membrane potential depolarisation was conveyed via a CCKB-dependent activation of Transient Receptor Potential (TRP)/TRPC-like channels in PCs of the BLA and entorhinal cortex (217, 222). In contrast, activation of CCKBRs in LVIb of the somatosensory cortex induces membrane potential depolarisation mainly via inhibition of K^+^ conductances (220) suggesting conserved and brain-area specific CCK effects.

### CCK-mediated behaviours

Initial research focusing on the GI regulation of food intake showed that (central and/or peripheral) administration of CCK resulted in a satiety response in animals (226, 227), but long-term administration of CCK as regulator of satiety proved unsuccessful in humans (228). Likewise, CCK coexpression in dopaminergic neurons (229, 230) spurred the hypothesis that CCK and dopamine functionally interact and that symptoms of schizophrenia could be alleviated by pharmacological intervention of the CCK-CCKR system. Unfortunately, drugs of the CCK-CCKR system did not deliver the hoped-for breakthrough in humans (231–234) (see **Table 3**).

**Table 3:** Role of selected neuropeptides/neuropeptide receptors in human mood disorders.

Receptors	Subtypes	Endogenous Ligands	Methodology	Behavioural effects	Comments
Cholecystokinin	CCK 1 CCK 2	Cholecystokinin	– Pharmacological intervention and subsequent behavioural assessment (231, 232, 234, 771–778) – Plasma and CSF analysis (779–781) – – *Postmortem in situ* hybridisation (782, 783) – Postmortem RT-PCR (784)	– Increased CCK signaling may be associated with panic disorders – Schizophrenic patients may exhibit decreases in CSF CCK levels as well as decreased CCK levels in the frontal and temporal cortex – *Postmortem* samples of depressed patients who have committed suicide show higher expression of CCKB-R in the prefrontal and cingulate gyrus	– Although a lot of studies showed preliminary efficacy for the treatment of depression, schizophrenia or anxiety, clinical trials were unable to corroborate these findings
VIP	VPAC 1 VPAC 2 PCA1	VIP	– Plasma and CSF analysis (293–295) – Genetic correlation analysis in patients with bipolar disorder (785)	– VIP plasma concentrations may be correlated with affective and anxiety disorders – Lithium decreases VIP plasma concentration in patients with bipolar disorder – SNP of the VIP gene are associated with BD In BD patients, lithium decreases CSF VIP levels	Most studies rely on a rather small number of participants
Somatostatin	SSTR 1 SSTR 2 SSTR 3 SSTR 4 SSTR 5	Somatostatin	– *Postmortem in situ* hybridisation (367, 368, 786) – CSF analysis (787, 788)	– Reduced SOM levels in PFC of depressed patients – Treatment of schizophrenics with fluphenazine and patients with affective disorder with nimodipine, decreases, respectively increases CSF SOM levels	–
Neuropeptide Y	NPY1R NPY2R NPY5R	Neuropeptide Y	– Plasma and CSF analysis (418, 419) – Pharmacological intervention and subsequent behavioural assessment (423) – – Pharmacological intervention and subsequent fMRI (789, 790) – *Postmortem in situ* hybridisation (421, 791), qPCR and ELISA (422) – Genetic correlation analysis (792–795)	– Reduced NPY in plasma of depressed patients (HPLC and radioimmunoassay). No changes were observed in CSF – Reduced NPY mRNA and increased NPY1R and NPY2R in the PFC and hippocampus of *postmortem* suicidal patients – Intranasal NPY administration led to no significant change in depressive and suicidal behaviour – Variation of NPY expression is associated with an altered salience network	– Concentrations for intranasal application were extrapolated and no dose ranging studies were conducted
Bombesin	NMB R GRPR BRS-3	Bombesin	–	–	– Up to date no studies were performed in humans
Orexin	ORX 1(A) ORX 2(B)	Orexin/ Hypocretin	– Pharmacological intervention and subsequent behavioural assessment (796–799) – Microdialysis analysis in amygdala (800) – Plasma analysis (801) – CSF analysis in MDD patients (546, 547) – RT-PCR analysis on blood cells of MDD patients (545)	– Orexin-B receptor antagonists show antidepressant as well as sleep promoting effects and have the potential of being used as adjunctive therapy in MDD – Acute orexin antagonism modulates anticipatory anxiety – Orexin-B receptor antagonist suvorexant discussed as preventive medication of delirium in hospitalized patients – Reduced orexin levels in the CSF of suicidal patients with MDD – Reduced orexin mRNA levels correlate with depression severity – Increased orexin levels in the CSF of MDD patients	–
Galanin	Gal1R Gal2R Gal3R	Galanin	– Pharmacologic intervention and subsequent behavioural assessment (802) – *Postmortem* qPCR and bisulfite pyrosequencing (803) – Genetic correlation analysis (804–808) Plasma analysis (809)	– *Postmortem* analysis shows differences in GAL receptor expression in depressed patients. While there was an increase of galanin and Gal3 receptor in the locus coeruleus and dorsal raphe nucleus in all subjects, only male subjects showed an increase of galanin and Gal3 receptor and a decrease of Gal1 receptor in the dlPFC. – Intravenous infusion of galanin shows a rapid anti-depressant effect – Genetic correlation with anxiety and depression. rs948854 polymorphism is associated with more severe anxiety pathology as well as depressive symptoms in females. This allele is further associated with more severe vegetative depressive symptoms and worse treatment response on antidepressants – Plasma levels may be a biomarker for severity of major depressive disorder	– Marked differences between human and rodent galanin system
Oxytocin	OXT	Oxytocin	– Intranasal application and subsequent behavioural assessment (810–830) – Intravenous application and subsequent behavioural assessment (831, 832) – Pharmacological intervention and subsequent fMRI (725, 814–816, 833–849) – Pharmacological intervention and subsequent EEG analysis (850–855) – Pharmacological intervention and subsequent eye-tracking (856) – Pharmacological intervention and subsequent neurophysiological monitoring with electrodermal electrodes (857, 858): – Genetic analysis of ASD patients (859)	– Intranasal oxytocin application: leads to prosocial effects and can potentially mitigate social deficits and other core symptoms (e.g. repetitive behaviour) in ASD (860) • may be an effective add-on for psychotherapy for in patients with severe depression or general distress • is shown to modulate activity of socially relevant brain regions (amygdala, OFC or superior temporal sulcus) • modulates the interplay between interoceptive and external salience processing • Chronic and acute application of oxytocin show differential effects. While acute oxytocin administration mainly affects neural activity and the oxytocinergic system, repeated administration decreases medial prefrontal N-acetylaspartate and glutamate-glutamine levels. These latter changes correlate with improvements in mPFC activity during a social judgment task • There is a possible link between metabolic syndrome, schizophrenia and oxytocin (861–863)	– There is a strong bias towards male participants in clinical trials – Studies performed in children show differential effects to those in adults – The net concentration of intranasally applied oxytocin may not be sufficiently high (864)
Vasopressin	V1A V1B	Vasopressin	– Intranasal application and subsequent behavioural assessment (865–870) – Pharmacological intervention and subsequent fMRI (871–876) – Plasma analysis (714, 877–879) – Genetic correlation analysis (880)	– Intranasal vasopressin: • Modulates risk-taking behaviour as well as willingness of cooperation during games with financial consequences (“Stag hunt” cooperation game) • Improves social deficits in children with ASD – Possible link to pathophysiology of depression (881, 882) – Plasma concentrations of arginine vasopressin tend to be elevated in depressed patients and veterans with PTSD and may reflect amygdala activation in men – Antagonism of V1A receptor shows anxiolytics effects – A SNP haplotype of arginine vasopressin 1B receptor protects against recurrent MDD	– Sex-dependent effects
Neurokinin	Neurokinin 1 Neurokinin 3	Substance P Neurokinin A Neurokinin B	– Pharmacological intervention and subsequent behavioural analysis (883–891) – Pharmacological intervention and subsequent PET (892–894) – *Postmortem* antibody staining (895) – *Postmortem* autoradiography (896) – Serum and CSF analysis (897–901)	– NK-1 receptor antagonism: • Showed no efficacy in treatment of social anxiety disorder, general anxiety disorder, PTSD, MDD or as an add-on to antidepressants • Receptor antagonism seems to modulate emotional processing in healthy individuals • NK-1 receptors were shown to be decreased in depressed rostral OFC and increased in *postmortem* schizophrenic PFC – Substance P: – Serum and CSF concentration does not show any correlation with depression severity, although increased concentrations were reported in serum and CSF in depression. However, patients with treatment resistant depressions showed consistently lowered concentrations.	– Lack of efficacy may be caused by insufficient receptor occupancy (902) Most of the studies were conducted with concentrations below 300 mg
Opioid	mu receptor kappa receptor delta receptor	(Met-/Leu-) Enkephalin Dynorphin	– Pharmacological intervention and subsequent behavioural assessment (903–913) – Pharmacological intervention and subsequent MEG (914, 915) – Pharmacological intervention and subsequent EEG (916, 917) Pharmacological intervention and subsequent fMRI (918) – Plasma analysis in depressed or schizophrenic patients (919–921) – *Postmortem in situ* hybridisation (922)	– Activation of opioid receptors attenuates depressive-like behaviours – Intranasal ketamine application has a fast antidepressant effect probably mediated via the kappa receptor and reversed by administration of naloxone. – The opioid system may play a role in the pathophysiology of schizophrenia since plasma mu and kappa receptors as well as endomorphin-2 are significantly increased in *postmortem* brains of schizophrenics – Buprenorphine seems to be suitable as a drug against major depressive and suicidal behaviour (923) – Patients with MDD show lower plasma kappa receptor levels. Selective kappa antagonism may be a suitable therapy for MDD	– Investigation of purely opioidergic effects are difficult since there is a major crosstalk between glutamatergic, dopaminergic and opioidergic signaling – Investigation of ketamine may be influenced by its ability to block glutamatergic receptors
CRF/CRH	CRFR 1 CRFR 2	CRF/CRH	– Pharmacological intervention and subsequent behavioural evaluation (924–929) – Plasma and CSF analysis (930–933) – Genetic correlation analysis (934)	– CRF receptor antagonists showed only limited effect in clinical trials – Depressed patients exhibit higher plasma CRH levels, CSF CRH levels as well as down-regulation of CRH receptor numbers in the cortex – CRH polymorphisms are not associated with the risk of MDD	– PTSD affects the HPA axis (935, 936)

ASD, autism spectrum disorder; BD, bipolar disorder; CSF, cerebrospinal fluid; dlPFC, dorsolateral prefrontal cortex; EEG, electroencephalography; ELISA, enzyme-linked immunosorbent assay; fMRI, functional magnetic resonance imaging; HPLC, high-performance liquid chromatography; MDD, major depressive disorder; MEG, magnetoencephalography; mPFC, medial prefrontal cortex; OFC, orbitofrontal cortex; PET, positron emission tomography; PTSD, post-traumatic stress disorder; qPCR, quantitative polymerase chain reaction; RT-PCR, reverse transcriptase polymerase chain reaction; SNP, single nucleotide polymorphism.

In the living animal, cortical CCK release correlates with the anticipation of social defeat and other stressors leading to anxiety-like behaviours that are – at least partly – mediated via CCKBR activation (149–151). Likewise, CCK administration to humans results in panic symptoms (149, 235). In agreement with this, central or peripheral administration of CCK-8 to male rodents reduces exploratory and social behaviour (236), possibly due to enhanced fear learning via CCKAR- and CCKBR-dependent facilitation of hippocampal long-term potentiation (LTP) and hippocampus-dependent spatial memory (237–239). In agreement with this, CCK-INs contribute to the maintenance of theta power and stable spatial representations in the hippocampus (240). In addition, CCK has been shown to promote LTP induction in the auditory cortex and to promote trace fear and delay fear conditioning (241–243). Somewhat in contrast to this, peripheral injections of CCK-8 impair the acquisition of active avoidance behaviour and facilitate the extinction of avoidance in rats (244, 245). Differences in dosage and in the CCK-CCKR system between mice and rats may partly explain these contrasting results. Nonetheless, these data indicate that CCK signalling contributes to behavioural changes in response to stressful stimuli and/or fear.

The role of CCK is not limited to spatial and fear memory as optogenetic inhibition of mPFC CCK-INs has been shown to impair the retrieval and use of working memory representations necessary for goal-directed behaviour (246).

In summary, animal studies clearly suggest that CCK modulates anxiety-like and fear-related behaviours, however, pharmacological studies in humans have so far failed to find efficient cures against schizophrenia, anxiety or MDD.

## VIP

### Discovery and expression in the brain

VIP was originally isolated from the porcine duodenum (247) and described as a vasoactive substance (248). It is a member of the secretin/glucagon superfamily of peptides and shares closest structural and functional homology with the pituitary adenylyl cyclase-activating peptide (PACAP) (249, 250). Other peptide members of this family are: Calcitonin, gastric inhibitory peptide (GIP), corticotropin-releasing hormone (CRH) and parathyroid hormone (251). VIP concentrations in the human cerebrospinal fluid (CSF) are ten times higher compared to the human plasma (252), which is why VIP has been suggested to serve as a neurotransmitter substance (253). In the PNS, VIP is mostly restricted to myenteric and submucosal neurons of the GI tract and to post-ganglionic fibres of the autonomic nervous system (210, 254). In the brain, strong VIP expression is observed in the cerebral cortex, suprachiasmatic nucleus (SCN) and medial nucleus of the amygdala (MeA). Lower levels are found in the hippocampus, the anterior pituitary and different hypothalamic nuclei (preoptic nucleus (PON), para- and periventricular nucleus) (255–257). In the rodent and human brain, VIP expression is mostly but not exclusively restricted to GABAergic INs (34, 37, 46, 257–261). In the PFC, VIP-expressing GABAergic INs are mostly located in supragranular cortical layers, however, a recent study suggests VIP expression in Betz cells of the marmoset primary motor cortex (261) and weak VIP mRNA in Betz cells of the human motor cortex (https://cellxgene.cziscience.com/gene-expression).

Ligand-binding studies initially showed high to moderate binding in the cerebral cortex, hypothalamus, amygdala, thalamus and hippocampus (262, 263) suggesting the existence of specific VIP receptors in the brain. The actions of VIP are mediated via three distinct G Protein-coupled receptors, named VPAC1-R (VIP1R), VPAC2-R (VIP2R) and PAC1-R, although the latter exhibits a lower affinity to VIP compared to PACAP (251, 264). In the rodent brain, VIP1R levels are particularly high in the cerebral cortex; the piriform area, the thalamus, and the hypothalamus (supraoptic nucleus (SON), mammillary body) and sensory and motor brain stem nuclei. VIP2R expression is high in the hippocampus, the periventricular zone of the hypothalamus, the cerebral cortex, sensory and motor brain stem nuclei, the amygdala and basal ganglia. PAC1R expression is altogether smaller compared to either VIPR. It is highest in LI of the cerebral cortex, the periventricular zone of the hypothalamus, and sensory brain stem nuclei (265). In the human brain, VIP1R mRNA transcripts are found throughout the cerebral cortex and in lower numbers also in subcortical structures (2, 4).

### Cellular and network actions of VIP

In agreement with their high expression in SCN neurons, VIP and VIP2Rs play a role in photic gating, circadian pacemaking and diurnal activity patterns in rodents (266–268). Similarly, VIP2R activation is necessary for a daytime-dependent neuronal activity state shift in SCN neurons and, possibly, also in cortical neurons (266, 268, 269).

The cellular mechanisms of VIP on neurons were to a large extent identified in electrophysiological or imaging studies. VIP has been shown to exert a differential effect on spontaneous and/or evoked firing in neurons and increases as well as decreases in firing have been reported (270–276). Different methodological approaches (direct iontophoretic application elicits immediate VIP actions on spontaneous firing whereas global bath perfusion induces excitability changes within 6-75 min (276, 277)) may partly explain these divergent results. In thalamic neurons, VIP attenuates slow intrathalamic rhythmic activity most likely by enhancement of the hyperpolarisation-activated cation conductance, I_h_ (274). The intracellular signalling cascades leading to increased AP discharge seem to differ in cell types and brain regions (270–276). In hypothalamic, cortical and hippocampal PCs, VIP inhibits Calcium-activated afterhyperpolarisation currents and increases Ca^2+^ transients promoting high-frequency burst-like discharges and glutamatergic synaptic transmission (270–272, 278, 279); the effects being mediated via activation of either VIP1Rs or VIP2Rs.

**Figure 6:**
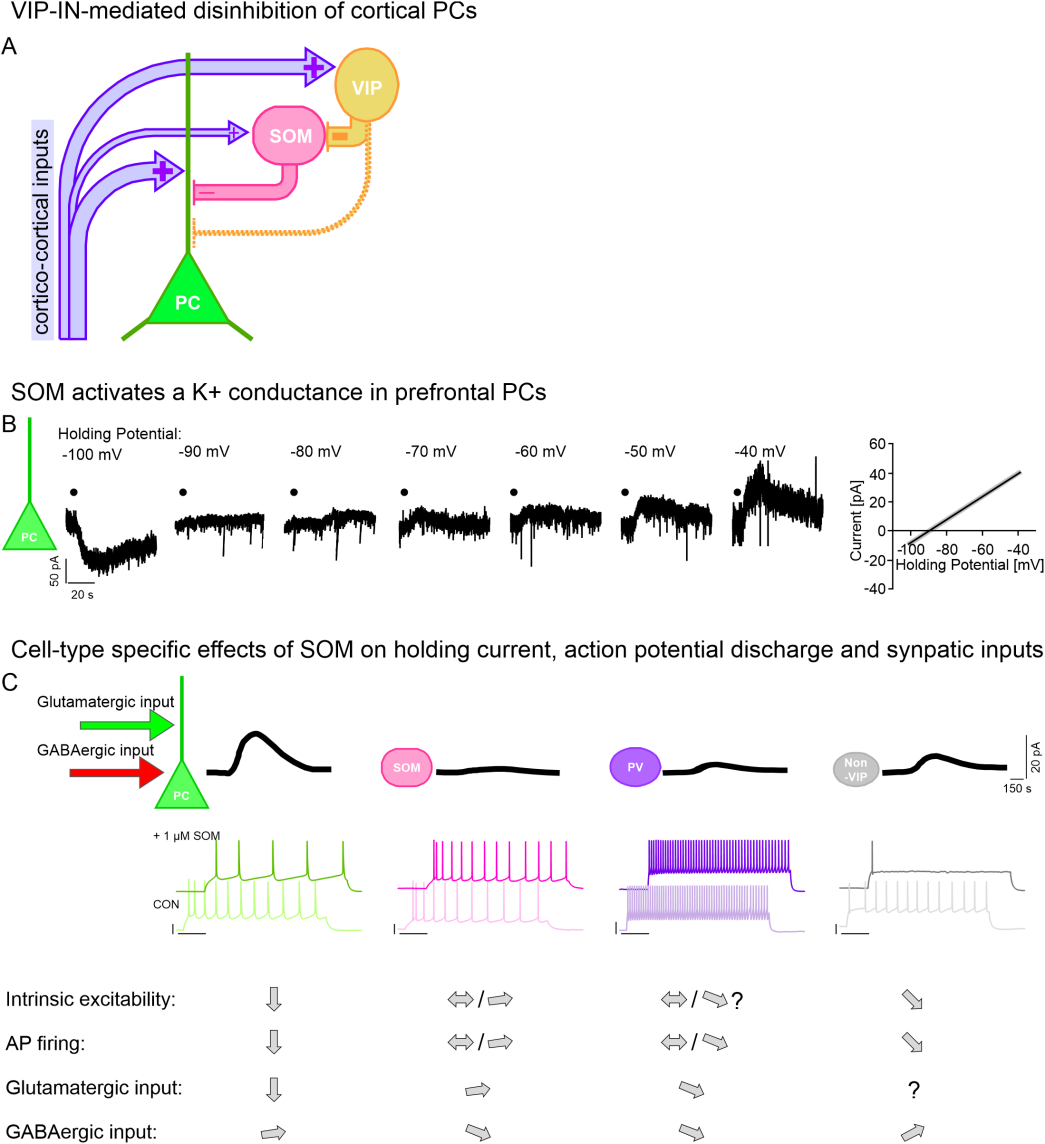
Neuropeptide-mediated effects on cortical processing the neuronal excitability. **(A)** Scheme showing VIP-IN-mediated PC disinhibition in response to cortico-cortical excitation. Cortico-cortical excitation preferentially reaches PCs and VIP-INs that in turn inhibit SOM-IN-mediated inhibition of PCs. **(B)** Whole-cell voltage-clamp recording showing exemplar current traces of SOM-induced holding current changes in response to different holding potential ranging from -100 to -40 mV in cortical PCs with corresponding illustration of SOM-dependent reversal potential according to (318). **(C)** Illustration showing that SOM induces cell-type specific effects on holding current (upper traces), evoked spiking (lower traces) and spontaneous glutamatergic and GABAergic inputs in different types of cortical/non-cortical neurons.

On a network level, VIP-INs of sensory cortex areas are primarily active during sensory input (45, 280–284) where they contribute to PC disinhibition by preferentially targeting SOM-INs (62, 63) (**Figure 6A**). Local field recordings in the awake mouse showed that deviance detection induces phase coherence between the ACC and the primary visual cortex (V1) at a peak of around 10 Hz, suggestive of top-down inputs from ACC to V1. Notably, VIP-INs contribute to this neuronal synchrony suggesting that VIP-INs distribute contextual information from higher cortical areas (285) thereby increasing responsiveness of V1 PCs to novel stimuli. Similarly, hippocampal CA1 VIP-INs together with CA1 PCs show increased activity during context- and object-related alterations in the environment suggestive of a general disinhibitory circuit motif of VIP-INs in different brain areas (286).

In addition, VIP-IN-mediated PC disinhibition by afferents from MD thalamic neurons possibly contributes to working memory by helping to maintain local excitation and supporting delay period activity in a delayed choice test (22, 287, 288). Further evidence for a possible role of VIP-INs in working memory has been provided by showing that specific dopamine receptor 1 activation on VIP-INs enhances working memory in the mPFC (289).

### VIP-mediated behaviours

It is undisputed that the rodent VIP-VIPR system is a potent modulator of neuronal activity. There is strong evidence that cortical VIP-INs help to encode the outcome of a behavioural output (reward versus punishment) (290) by contributing to sensory processing and working memory. In agreement with such hypothesis, VIP-IN activity reduces food intake by modulating the salience of, value of, and/or attention to a given food source (291). In addition, VIP-INs guide open arm avoidance behaviour in the elevated platform maze (EPM) test by enhancing prefrontal response to ventral hippocampal inputs during open arm exploration (292). These studies collectively support the role of VIP in guiding aspects of goal-directed behaviours.

On the basis of rodent studies, it was suggested that VIP levels in humans may be correlated with affective or anxiety disorders (293–295) but initial results need to be validated by a larger patient cohort.

## Somatostatin

### Discovery and expression in the brain

In the late 60s and early 70s of the last century, crude extracts from different hypothalamic areas were shown to increase or decrease the amount of growth hormone (GH) released by pituitary cells (296–298). The release-promoting factor of GH was named GH-releasing hormone (GHRH), the release-inhibiting factor was named somatotropin release-inhibiting factor (SRIF) or somatostatin (SOM). The biologically active form of SOM consists of a 14- and a 28 amino acid-long peptide, both of which are expressed in the brain (299, 300). In the years following its discovery, SOM was found to not only block the release of GH but also that of gut-associated hormones such as insulin, glucagon, CCK and secretin (185–192). Under physiological conditions, SOM is released into the median eminence following activation of neurons from the periventricular and paraventricular nucleus of the hypothalamus. The daily release of SOM follows a pulsatile fashion (134) and is promoted by food deprivation and other stressors (145–148). In addition, SOM was shown to be widely expressed by cortical neurons (300, 301), often together with neuropeptide Y (NPY) (302–304). We now know that the majority of these cortical SOM-expressing neurons are GABAergic INs (304–306), whereas SOM receptors are found on PCs as well as on GABAergic INs (307–309). Ever since its discovery in the cortex, SOM was hypothesised to “function as a neurotransmitter in areas of the brain remote from the median eminence” (300). Indeed, Renaud et al. (1975) proved the existence of SOM-responsive neurons in the cerebellar and cerebral cortex (310). In addition, studies suggest local SOM release by hippocampal and cortical neurons (174–178, 311).

The biological effects of SOM are mediated via activation of five different subtypes of seven-transmembrane domain G protein-coupled somatostatin receptors (SSTR1-SSTR5). Ligand binding to SSTRs triggers a G_i_ protein signalling cascade that results in inhibition of adenylyl cyclase and thus reduces intracellular cyclic AMP levels. In addition, the G_β γ_ subunit of SSTRs interacts with inwardly rectifying potassium (GIRK) channels and promotes their opening (312–315). The determination of which SSTR conveys which physiological effects has been partially incongruent, likely due to tissue-specific expression levels of SSTR subtypes: *In-situ* hybridisation, immunocytochemical and transcriptomic studies show the presence of SSTR-1, SSTR-2 and SSTR-3, SSTR-4 signal throughout the rodent and human brain, however there are regional variations in SSTR subtype mRNA levels (1, 3, 4, 307–309, 316). Single-nucleus RNAseq analysis of human PFC neurons shows highest amounts of SSTR-2 mRNA levels, followed by SSTR-1 and SSTR-3 mRNA levels in excitatory and inhibitory neurons alike, SSTR-4 and SSTR-5 transcripts being very weak to virtually absent in either cell type (4). It could further be shown that SSTR-2 and SSTR-3 mRNA transcripts are present in the majority of IT PCs, whereas SSTR-4 mRNA levels are higher in PT and CT PCs of the mouse visual and anterior lateral motor cortex. In contrast, SSTR-1 mRNA transcripts are preferentially present in different types of SOM-INs (1).

Electrophysiological and imaging studies on cortical, basal forebrain, thalamic, brain stem and amygdalar neurons and cortical astrocytes using specific SSTR agonists and/or specific SSTR antagonists suggest that SOM exerts its effects mainly via activation of SSTR-2 (163, 317–325). In contrast, electrophysiological studies in hippocampal neurons suggest modulation of neuronal activity mainly via activation of SSTR-4 (176, 323, 326, 327) or SSTR-2 (328, 329).

### Cellular and network actions of SOM

Studies on cortical and non-cortical cells majorly report that SOM activates different K^+^ channels, e.g. GIRK and Kv_7_ channels, to induce membrane potential hyperpolarisation and an inhibition of spontaneous and/or evoked firing in hypothalamic, hippocampal, entorhinal, striatal, amygdalar, cortical, brain stem and pituitary cells (176, 310, 315, 318, 319, 322, 326, 330–342) (**Figure 6B**). These studies give important mechanistic insights into the physiological effects of exogenous SOM. The effect of endogenously released SOM on PCs of the mPFC was tested by optogenetic stimulation of channelrhodopsin-2-expressing SOM-INs of the SST-IRES-Cre mouse in the presence of blockers of fast and slow GABAergic transmission: Under this condition, optogenetic stimulation resulted in membrane potential hyperpolarisation in roughly one third of PCs (330). In the entorhinal cortex, optogenetic stimulation of SOM-INs results in a prolonged, (mostly) SSTR4-dependent inhibition of spontaneous AP firing in PCs while optogenetic activation of PV-INs or of SOM-INs in a SSTR4-KO mouse provides only a transient inhibition of AP firing (176). Additional electrophysiological data suggest that SOM acts differentially on distinct cortical neuron types by inducing a significantly larger outward current and inhibition of evoked firing in PCs compared to subtypes of GABAergic INs (163, 319) (**Figure 6C**). It remains to be tested how other IN types such as CCK- or VIP-INs respond to SOM as a SOM-induced hyperpolarisation in non-PCs was observed in the PL cortex (330).

In addition, SOM has been shown to inhibit voltage-dependent Ca^2+^ channels in retinal, thalamic, hippocampal, amygdalar, striatal and sympathetic neurons (319, 323, 324, 343–347).

Above-mentioned inhibition of voltage-gated Ca^2+^ channels by SOM leads to a presynaptic inhibition of glutamate (319, 323, 324, 328, 348–350) and GABA (319, 323, 325, 351, 352) release in neurons of the striatum, cortex, thalamus, hippocampus, brain stem and spinal cord. The effect of SOM on glutamatergic transmission is predominantly conveyed via afore-mentioned presynaptic block of glutamate release and a postsynaptic de-excitation of PCs but not via a direct postsynaptic inhibition of ionotropic glutamate receptors. The SOM-dependent reduction of excitatory inputs affects PCs as well as PV-INs but not SOM-INs, whose excitatory drive is increased in a GABA_A_R-dependent fashion in response to SSTR activation (353) (**Figure 6C**). Owing to SOM’s differential effects on distinct IN classes, the modulation of GABAergic transmission is a little more complex. At the presynaptic level, SOM does not alter or slightly increases spontaneous GABA_A_-R-mediated transmission onto PCs, local striatal INs and LI INs but decreases that onto striatal medium-spiny neurons and cortical SOM-INs (319, 352) (**Figure 6C**). At the postsynaptic level, SOM does not modulate GABA_A_-receptor-mediated currents but inhibits GABA_B_-receptor mediated currents in apical dendrites of cortical PCs (319), suggesting an interaction between SSTRs and GABA_B_Rs, possibly with the aim to control presynaptic SOM and/or GABA release (177) and to reorganise PC inhibition (354). On a network level, the overall divergent effects of SOM on PCs and SOM-INs increase correlated activity between PCs and decrease that between PCs and SOM-INs (319, 324). Loss of correlation between SOM-INs and PCs has also been observed during a visual discrimination task in V1 of mice and this de-correlated activity between SOM-INs and PCs allows the gating of task-related, top-down information in LI (355, 356).

### SOM-mediated behaviours

On a behavioural level, increased SOM-IN activity is correlated with PFC-dependent exploratory (330, 357) and risk assessment behaviour (358), both of which might contribute to SOM-IN-dependent facilitation of LTP induction and encoding of fear memory (352, 359–361). In addition to modulating exploratory behaviour and fear, SOM-INs are also implicated in affective state discrimination by exhibiting disproportionately increased activity in mice presented with a neutral mouse or with a mouse having just received a relief stimulus (water after 23 h of water deprivation) or a mild stress stimulus (immobilisation stress) (362). Moreover, SOM-INs have been shown to contribute to working memory performance (363–365).

As mentioned above, SOM mediates its effects partially by increased activity of the Kv_7_ channel and daily doses of the Kv_7_ channel opener ezogabine in MDD patients improve symptoms of anhedonia and depression, possibly by modulating brain activity between the cingulate cortex and striatum (366). Further evidence in support of a possible contribution of SOM(-INs) to MDD is provided by human *postmortem* studies showing that SOM mRNA levels in the ACC of depressed patients are significantly reduced (367, 368). This hypothesis is supported by a study showing that disinhibition of SOM-INs results in an anxiolytic and anti-depressant phenotype in mice (369).

Collectively, these data suggest that SOM-INs may be important regulators of mPFC-dependent cognitive tasks, however, it remains to be proven to what extent endogenously released SOM contributes to these effects and whether pharmacological interventions of the SOM-SSTR system are able to treat neuropsychiatric disorders (370) (**Table 3**).

## Neuropeptide Y

### Discovery and expression in the brain

NPY is a member of the family of pancreatic hormone polypeptides (PP) and consists of 36 amino acids. The presence of a chemically related peptide to PP, was first described in 1979 in the rat brain (371). In 1982, the amino acid sequence of this peptide was identified, the peptide was named NPY (372, 373) and its presence in the brain confirmed (374). Outside the brain, NPY has been shown to act as a vasoactive substance potentiating the effects of vasoconstrictor agents such as (nor-)adrenaline (375, 376). In the brain, NPY was shown to increase food intake in rats by acting on NPY-responsive neurons within the lateral hypothalamus (LH), the paraventricular and ventromedial nucleus of the hypothalamus (377, 378), the arcuate nucleus being the major source of NPY within the hypothalamus (379). Outside the rat hypothalamus, most NPY-reactive cell bodies are found in the cortex, the striatum and the hippocampus (374). Similarly, high amounts of NPY mRNA transcripts/NPY peptides have been identified in the human brain with a laminar expression pattern (1, 4, 380). Typically, highest NPY levels are found in the infragranular cortical layers (374), often in combination with GABA and SOM (34, 35, 381, 382).

### Cellular and network mechanisms of NPY

In the rodent brain, the biological actions of NPY are mostly mediated via activation NPY1, NPY2 or NPY5 receptors (NPY1R, NPY2R, NPY5R) that primarily couple to G_i/o_ proteins (383). Unlike SOM however, NPY neither increases K^+^ conductances nor does it reduce the input resistance of hippocampal and other neurons (384–387), but like SOM, it has been shown to reduce voltage-gated Ca^2+^ currents in neurons of different regions (388–392).

Electrophysiological studies showed that NPY inhibits excitatory synaptic transmission in cortical and other neurons (384–386, 393–396). This effect is mediated by a presynaptic inhibition of glutamate release and not by a postsynaptic inhibition of ionotropic glutamate receptor currents (384, 393). Additional studies later identified NPYR2s as mediators of NPY-dependent inhibition of glutamatergic transmission onto PCs and CCK-INs (397–401). In the cortex, the presynaptic inhibition of glutamate release by NPY is partly mediated by NPYR1s (402). NPY-dependent inhibition of glutamate release furthermore suppresses epileptiform activity in acute hippocampal slices mostly via activation of NPY2Rs (397, 403–405) and measurements of hippocampal NPY levels support the hypothesis of a local NPY source (406, 407). Given that loss of NPY-IN-mediated GABAergic inhibition of PCs increases superficial layer excitability in an NPYR1-dependent fashion and leads to slow spike and wave discharges associated with epileptiform activity (408), it is likely that local NPY-INs represent the major NPY source outside the hypothalamus.

In addition, there is evidence that NPY inhibits some aspects of GABAergic transmission onto hippocampal CCK-INs but not onto PCs (384, 401). In the cortex of young rats (P14-21), NPY exerts a dichotomous effect on inhibitory neurotransmission such that inhibitory inputs onto PCs are enhanced whereas those onto fast-spiking and low-threshold regular spiking INs are inhibited. Further, excitatory neurotransmission onto PCs is reduced following NPY exposure (385). Interestingly, NPY does not modulate glutamatergic nor GABAergic synaptic transmission in older animals. Rather, NPY suppresses Ca^2+^ conductances in the distal apical dendrites of LV cortical PCs resulting in a dynamic inhibition of synaptic plasticity and in a possible shift in the integration of top-down versus bottom-up inputs depending on the state of PC (387).

### NPY-mediated behaviours

*In vivo* studies suggest a role for NPY in anxiety, stress disorder and depression: Microinjections of NPY into the BLA or hippocampus reduce fear-potentiated startle and anxiety and promote long-term resilience to acute restraint stress-induced reductions in social behaviour (396, 409–411). Similarly, NPY over-expressing rats present with insensitivity towards stress and absent fear suppression (412) and NPYR1 over-expressing mice show a modest anxiolytic-like phenotype in the open field (OF) and the EPM test (413). In contrast, NPY KO mice tend to exhibit anxiogenic-like phenotypes (414). The effects of NPY on fear and/or anxiety seem to be mediated by a combined activation of NPY1R and NPY2R (415). In addition to modulating fear and/or anxiety, i.c.v. administration of NPY promotes anti-depressant-like effects in the forced swim (416). Likewise, central administration of NPY promotes stress resilience in a rat model of post-traumatic stress disorder (PTSD) (417).

Studies in humans point to a similar role of NPY in stress and/or mood disorders such that NPY plasma levels tend to be reduced in depressed and PTSD patients (418–420). Moreover, *postmortem* studies suggest significantly decreased NPY (mRNA and protein) expression in the PFC of depressed suicides (421, 422). In light of these and related studies, NPY was tested as a possible antidepressant, however, no sustained improvements were observed (419, 423) (**Table 3**).

## Bombesin

### Discovery and expression in the brain

Methanol extracts of the skin of two Bombina frog species were shown to exert a pharmacological action on vascular and extravascular smooth muscles. This finding led to the isolation of the suspected pharmacological agents and their amino acid sequence determination. One of the substances was hence named bombesin (424–426). It conveys its actions via its C-terminal octapeptide (427).

In mammals, two bombesin-like counterparts have been identified and characterised: Gastrin-releasing peptide (GRP) and neuromedin B (NMB) (428). Endogenous bombesin-like peptide expression in the rat brain was confirmed by immunochemistry on micropunches of tissue and immunocytochemistry on rat brain slices (429–434). Ligand-binding studies later revealed at least two distinct bombesin binding sites with high binding sites in the hypothalamus, hippocampus, amygdala and FC (431, 435). These binding sites differ in their relative affinity to either GRP or NMB (436, 437) and both peptides display distinct expression profiles: GRP mRNA is mostly found in the SCN and SON, in the hippocampal formation, the amygdala and the cortex whereas NMB mRNA is mostly restricted to the olfactory regions, the substantia nigra (compact part), the ventral tegmental area (VTA) and the trigeminal and dorsal root ganglions (438, 439). The GRPR mRNA profile partly follows that of GRP: NMB receptor mRNA transcripts are mostly confined to the thalamus (central medial and central lateral nucleus). Protein levels of GRPR have also been reported in the mouse ACC, the primary motor cortex (M1), V1, the primary somatosensory (S1) and primary auditory cortex (A1), the insula, the hippocampus and the amygdala, substantially overlapping with GAD67 immunoreactivity (440–443). In the human brain, GRPR mRNA transcripts/peptides are found at high density in the hypothalamus, but also in the PAG, the striatum, the hippocampus, the septum, the amygdala and the PFC (2, 4, 444).

Based on sequence similarity, a third bombesin receptor subtype (BRS-3) was identified, however, this receptor does not display high affinity to any known bombesin-like peptides and is thus considered an orphan receptor (445, 446). Immunolabelling for BRS-3 in the rat brain revealed high immunoreactivity throughout the brain (447).

### Cellular and network actions of bombesin

Early work suggested bombesin as a modulator of distinct behavioural outputs following the observation of bombesin-like peptides release from central neurons of the rat (448, 449). Electrophysiological studies helped to understand the cellular mechanisms of action. Bombesin and/or GRP-responsive neurons have been reported in the PON and arcuate nucleus (450, 451), the nucleus raphe (452, 453), the hippocampus (454, 455), the entorhinal cortex (456), the amygdala (442), A1 (443) and the ACC (440). Bombesin and/or GRP induce(s) an inward current and/or a decrease in membrane potential. This decrease in membrane potential in serotonergic cells of the nucleus raphe and in GABAergic of the PON, of the hippocampus or of the entorhinal cortex is accompanied by increases in spontaneous spiking and by decreases in K^+^ conductances and/or increases in cationic conductances (Na^+^, Ca^2+^). In the ACC, the amygdala and the hippocampus, GRP-mediated excitation of GABAergic INs produces a subsequent increase of GABAergic but not glutamatergic synaptic transmission onto PCs (440, 442, 454). In many cortex areas, GRP-responsive GABAergic cells are mostly VIP-INs. In A1, their GRP-dependent excitation promotes PC disinhibition by enhanced inhibition of SOM-INs (443). Given the fact that VIP-INs are suggested to encode aversive cues (62, 457), it is inferred that GRPRs enhance conditioned fear memory by recruiting disinhibitory circuits in the auditory cortex (443). In this context, GRPR KO mice exhibit an increased fear memory and microinjection of GRP into the IL cortex or the BLA reduced freezing in a conditioned fear paradigm (458, 459).

### Bombesin-mediated behaviours

In addition, injections of bombesin via different routes of administration (i.c.v., intrathecal, i.p.) caused compulsive grooming behaviour (460–463) that was independent of adrenal or hypophyseal activity (464) but under the influence of dopaminergic, GABAergic or kappa opioidergic activity (435, 465–469). Similarly, direct injections of bombesin into the nucleus accumbens were shown to induce grooming behaviour (467). We now know that activation of GRPRs on secondary itch afferents may partly explain bombesin-induced grooming behaviour in rodents (470). To date, it is unknown to what extent pharmacological manipulation of the bombesin-GRPR system may modulate compulsive behaviours in humans.

Moreover, bombesin plays an important role in energy homeostasis: (1) Peripheral or central administration of bombesin increases satiety and reduces food intake in rodents and humans (471–476). This effect seems to be specifically mediated via a hypothalamic action (435, 474, 477–479). In GRPR-deficient mice, the bombesin-mediated suppression in food intake is reduced suggesting that the satiety response is – at least partially- mediated via GRPR (480). (2) I.c.v. or intracisternal injection of bombesin or GRP cause a rapid decline in body temperature in rats (481–483), the effect most likely being mediated by bombesin-responsive neurons in the PON (484, 485). (3) Despite its classification as orphan receptor, BRS-3 deficient mice are reported to develop obesity, reduced metabolic rate and increased food intake (486–488). These findings are supported by the fact that activation of BRS-3 receptors by a selective nonpeptide BRS-3 agonist (compound A) enhances energy expenditure and decreases food intake in rats (489, 490). Similarly, optogenetic activation of BRS-3-expressing neurons in the dorsomedial hypothalamus of mice increases body temperature by stimulating neurons in the raphe pallidus that induce sympathetic activation of brown adipose tissue and increases in heart rate. In contrast, activation of BRS-3-expressing neurons of the paraventricular nucleus of the hypothalamus reduces food intake, suggesting a specific role for hypothalamic BRS-3 expressing neurons in energy homeostasis (491).

In summary, animal studies clearly show a relationship between GRPR/BRS-3 and grooming behaviour and energy homeostasis, respectively. Clinical trials in humans with an orally active BRS-3 agonist partially support the findings of bombesin on energy homeostasis but failed to prove as efficient anti-obesity treatment as no significant effects on hunger sensations/food intake were reported (492).

## Orexin/Hypocretin

### Discovery and expression in the brain

Hypocretins were identified in the posterior hypothalamus as a family of the incretin hormone family (493). In an independent study of the same year, two endogenous ligands for multiple orphan receptors were identified in the LH. Central administration of these ligands to rats stimulated food intake, hence the name orexin. Hypocretins/orexins mediate their actions via two distinct receptors, named orexin A (Hcrt-1) and orexin B (Hcrt-2), respectively. Orexin A receptor has a higher affinity to orexin A whereas orexin B receptor has a higher affinity to orexin B. Orexinergic (ORX) neurons of the hypothalamus receive inputs from prefronto-cortical neurons and to a higher degree from other hypothalamic nuclei (e.g. PON and SON, arcuate nucleus), from the central and basomedial nucleus of the amygdala (CeA, BMA, respectively), from the basal forebrain or from brain stem nuclei such as the raphe nuclei or the vagal nucleus (494).

ORX fibres are found in numerous brain regions including the cortex, the amygdala, the septum, the thalamus, the hypothalamus, the midbrain and the brain stem (495–497). In the PFC, the distribution of ORX fibres exhibits a rostro-caudal gradient with increasingly more fibres towards the caudal part of the mPFC, the majority of fibres projecting ipsilaterally to the mPFC (498, 499). Dense orexin A receptor expression is found in the rat olfactory system, in the bed nucleus of the stria terminalis (BNST), in thalamic (anterodorsal, centrolateral, reticular, ventral posterior, zona incerta) and hypothalamic nuclei (arcuate, paraventricular, periventricular, supraoptic, suprachiasmatic, ventromedial) and in certain brain stem nuclei (e.g. LC or olivary complex) (500, 501). The rat PFC exhibits moderate expression levels of orexin A receptors (501, 502) with most orexin A receptor-expressing cells present in LII/III (498). In addition, functional evidence for the presence of orexin B receptors in the mPFC has been provided (503, 504). Similarly, mRNA transcripts for orexin A and orexin B receptors have been shown in the human PFC (4).

### Cellular and network actions of orexin/hypocretin

In thalamic paraventricular projection neurons, orexin A and orexin B administration depolarises the membrane potential, increases spontaneous AP discharge by blocking a K^+^ conductance and the slow afterhyperpolarisation conductance (505), the effect of orexin B being significantly larger compared to orexin A (497, 506, 507). Similar orexin actions were described in thalamic centromedian or rhomboid neurons but not in neurons of the ventro-posterolateral (VPL) thalamic nucleus (508), consistent with a lack of orexin A or orexin B receptor expression in the VPL thalamic nucleus (501). In cultured SCN neurons, orexin A administration induces increased spontaneous spiking in 38% cells and decreased spiking in 28% of cells with around 33% not responding to orexin A. In addition, orexin A via activation of orexin A receptors has been shown to inhibit spontaneous inhibitory synaptic transmission in SCN neurons and to cause an advance phase shift in the circadian activity profile of dissociated and organotypic SCN neurons (509). Similarly, orexin A or B lead to membrane potential depolarisations and increases in spiking and excitatory but not inhibitory synaptic transmission in hypothalamic and non-cortical neurons (508, 510–515).

In VTA dopaminergic neurons, orexin A potentiates evoked NMDAR-, and more specifically NR1/NR2A-mediated currents without altering that of AMPARs. In addition, long-term ORX treatment causes an increase in spontaneous glutamatergic synaptic transmission and increases the AMPAR/NMDAR ratio and is suggested to block cocaine-associated plasticity, thereby modulating addiction-dependent synaptic plasticity and/or behaviour (516). In hippocampal DG neurons, orexin A potentiates field excitatory postsynaptic potentials (fEPSPs) (517) whereas degeneration of ORX neurons leads to reduced LTP induction in the CA1 region of the hippocampus (518). In line with this, ORX treatment has been shown to restore hippocampus-dependent memory in ORX-deficient mice (519).

In the PFC, orexin A excites supragranular PCs via an enhancement of a Na^+^ and inhibition of a K^+^ conductance (520, 521) and/or via blockage of HCN channels (522). Further, orexin A increases spontaneous glutamatergic synaptic transmission in LV PCs (523). Lastly orexin A enhances evoked and spontaneous GABAergic synaptic transmission onto prefrontal PCs of juvenile mice by facilitating presynaptic GABA release, likely from SOM-INs (524). Consistent with that, blockage of orexin A receptors leads to decreased spontaneous activity in infragranular PCs and to decreased gamma power (525). It is further suggested that ORXs act via orexin B receptors to trigger glutamate release onto LV PCs in the ACC, the effect being antagonised by the mu opioid agonist DAMGO (504). In neurons of the CeA, endogenous orexin A release or exogenous orexin A administration enhances spontaneous AP firing and induces anxiolytic-like behavioural effects and locomotor activity (526–529). In agreement with this, ORX KO mice exhibit increased anxiety-like behaviours (530). These data collectively suggest that ORXs increase the synaptic output of ORX-responsive neurons which may promote experience-dependent anxiolytic effects.

### Orexin/Hypocretin-mediated behaviours

On a network level, so-called hedonic hotspots in the rostromedial orbitofrontal cortex (OFC) amplify the hedonic impact of sweetness via activation of mu-opioid or orexin receptors (531). In agreement with this, morphine-cued place preference was increased following intra-PL cortex administration of the orexin A receptor antagonist SB334867 (532). Further, the connectivity between mPFC and LH_ORX_ neurons is necessary for orexin A receptor-mediated, mPFC-dependent cue-potentiated feeding in rats whereas disruption of this connectivity or blockage of orexin A receptors impairs cue-potentiated feeding (533).

Evidence has also been provided that orexin A receptors play a role in cost-benefit decision-making such that orexin A receptor inactivation in the OFC significantly decreases the rat’s preference for high food reward in a delay-based decision-making task (534), possibly via a crosstalk between galaninergic and ORX neurons (535). There is further evidence for a crosstalk between the ORX and the ghrelinergic system as ORXs or orexin receptor antagonists are known to modulate ghrelin-induced feeding in rodents (536–539).

Furthermore, ORXs may enhance spatial working memory in a delayed working memory task (non-matching-to-place T-maze task) and in spatial recognition (540).

Studies further suggest a link between the ORX system and (anti-) depressive-like behaviours such that hyper- or hypoactivity of the ORX system may contribute to a depressive-like phenotype. Studies in support of an anti-depressive function of orexins report that orexin A, possibly by acting on orexin-responsive neurons in the mPFC, induces anti-depressive like behaviours (reduced immobility time in FST) in mice (541, 542). Studies in support of a depression-promoting action of ORXs show that antagonism of orexin A and B receptors ameliorates CUS-induced depressive-like behaviours in mice (543). In addition, *in vivo* Ca^2+^ imaging of LH neurons shows increased activity during anxiety (EPM, OF test), despair (forced swim test, tail suspension test) and anhedonia (sucrose splash test). In addition, mice that have been subjected to CUS for 21 days exhibit upregulated c-fos levels and increased PC excitability in the mPFC as a direct result of increased excitability of LH_ORX_ neurons projecting to the mPFC. Optogenetic activation of the LH_ORX_-mPFC pathway on the other hand, induces anhedonia in an orexin A receptor-dependent fashion but not anxiety or despair in un-stressed mice (544). A different stress paradigm (20 min immobilisation stress for 7 consecutive days) results in decreased ORX-induced spontaneous glutamatergic transmission in coronal brain slices containing the PFC, a likely result of atrophy of the apical dendrites of LV PCs (523). Different methodological approaches (different stress paradigms, different electrophysiological readouts (excitability versus spontaneous excitatory synaptic transmissions)) do not allow a direct comparison of these findings as spontaneous excitatory synaptic transmissions was not monitored in the first CUS model.

These partially conflicting studies in rodent animals are also mirrored in human studies showing that either reductions or increases in ORX levels are correlated with a depressive phenotype/severity of depressive symptoms (545–547).

Despite partially conflicting results, these studies collectively show that ORXs may modulate goal-directed behaviour and contribute to MDD and depressive-like behaviours in humans and animals, respectively. Research on ORXs developed dual orexin A/orexin B receptor antagonists (DORAs) that have successfully progressed into human trials (548–552). To date DORAs are mainly prescribed for the treatment of insomnia and results on DORAs for the treatment of MDD still warrant further research (**Table 3**).

## Galanin

### Discovery and expression in the brain

Galanin is a 29-30 amino-acid-long neuropeptide that was originally isolated from the porcine intestine (553). Further work revealed that galanin expression was not restricted to the GI tract but was also present in the CNS. Galanin-expressing neurons are either excitatory or inhibitory and are found throughout the rodent brain with high densities in different hypothalamic nuclei but also in the septal region, the hippocampus and the PFC (554–559). With the help of autoradiography, galanin binding sites were demonstrated in most areas of the rat brain (560, 561). Later studies in humans and rodents revealed that galanin exerts its action via activation of three G protein-coupled receptor subtypes named Gal1, Gal2 and Gal3 receptor (Gal1R, Gal2R, Gal3R) (562–570). These receptors are distributed in a partly overlapping and partly distinct fashion. Roughly, Gal1R mRNA transcripts are preferentially, not exclusively, observed in the caudal rat brain (midbrain, brain stem), whereas Gal2R mRNA transcripts can be preferentially observed in the rostral brain with high transcript numbers in the olfactory system (2, 571). The same rostro-caudal gradient of Gal1R and Gal2R mRNA transcripts applies to hypothalamic nuclei (571, 572). In the mouse brain, this rostro-caudal gradient of Gal1R mRNA transcripts appears weaker (573). Only very limited Gal3R mRNA transcript levels can be observed in the rat brain, most of these in hypothalamic or brain stem nuclei (567, 569, 574).

Galanin synthesis – and possibly release – underlies a dynamic control in response to electroconvulsive stimulation (575), to peripheral (576, 577) or central (578) nerve injury or to other CNS lesions or manipulations (579–582). Similarly, exposure to different stressful stimuli (tail suspension, predator odour, intruder paradigm) increases the activity of galanin-expressing neurons of the LH (583). Indirect evidence suggests that galanin may be co-released with other transmitters (e.g. noradrenaline, NPY, serotonin, dopamine) from secretory granules in brain stem or hypothalamic neurons (584–589).

### Cellular and network actions of galanin

Galanin and specific galanin receptor agonists have been shown to exert mainly inhibitory effects on postsynaptic target cells of various brain areas by increasing K^+^ conductances and inhibition of spontaneous spiking (585, 590–597). These effects are partially mediated by co-activation of GABA_B_ receptors and rely on extracellular Ca^2+^ but occur independent of muscarinic receptor activation (596, 598). In addition, galanin has been shown to inhibit Ca^2+^ channels in peripheral and central neurons (599, 600) which may lead to an inhibition in presynaptic neurotransmitter release (601–604).

Noteworthy, the postsynaptic effects of galanin appear to be state-dependent such that short-term dehydration in rats induces a larger K^+^ conductance in SON neurons compared to non-dehydrated animals (605) and it is currently not known whether changes in osmolality result in similar differences in other hypothalamic nuclei or whether this observation is specific to SON neurons to control plasma osmolality.

In LC neurons, galanin effects appear to be mediated via activation of Gal1R whereas in other brain areas (e.g. SON, arcuate nucleus) these effects are mediated via (co-)? activation of Gal1R and Gal2R/Gal3R (591, 595, 602, 605).

The generally de-exciting actions of galanin suggest anti-convulsant properties. In agreement with this hypothesis, galanin KO and Gal1R KO mice develop spontaneous seizures associated with secondary generalisations and occurrence of spike-and-slow wave complexes, a likely result of reduced inhibitory transmission as the intrinsic excitability of CA1 hippocampal cells was not altered (606, 607). In contrast, galanin-overexpressing mice show less sustainable generalisations (608). Similarly, *in vivo* recordings in anaesthetised rats showed that galanin administration reduces the amplitude and speed of high potassium-induced cortical spreading depolarisations (CSDs) in a mostly Gal2R-dependent fashion and also reduces regional cortical blood flow (557, 609).

Collectively, these studies suggest that galanin dampens network activity and may inhibit epileptiform brain activity.

### Galanin-mediated behaviours

Studies in rodents report that galanin may modulate learning and memory processes. Studies either suggest galanin-dependent impairments or improvements of long-term changes in synaptic transmission or no alterations thereof. In summary, galanin is reported to impair LTP induction in hippocampal CA1 apical dendrites with no impairments of LTD (601, 610). Similarly, galanin or the non-peptide GalR agonist galnon impairs LTP induction in mouse DG in a Gal2R-dependent fashion (611, 612). In agreement with these findings, galanin infusions into the ventricles or the hippocampus impair spatial memory acquisition and/or retention and/or consolidation tested in the Morris water maze task, radial maze paradigm or EPM task (613–619). In contrast, field recordings in the entorhinal cortex report no alterations in the induction and maintenance of LTP, de-potentiation, LTD and muscarinic receptor-induced LTD in galanin KO mice. Nonetheless, a loss of spatial memory in a novel object location task is observed in 10-14 months-old galanin KO mice while cortex-dependent novel object recognition remains unaffected suggesting that loss of galanin impairs spatial, but not recognition memory (620). Likewise, young Gal3R KO mice (3 months) exhibit impaired spatial learning in the Morris Water maze. Interestingly, this impairment does not persist in 12-14 months-old Gal3R KO mice (621). No or only subtle galanin effects on spatial learning are observed in novel object discrimination after i.c.v. infusion of galanin or intranasal infusion of the specific Gal2R agonist M1145 (622, 623).

In addition, microinfusions of galanin into the medial septal area, the ventricles or the hippocampus result in working memory impairments as evidenced by a decreased choice accuracy in a delayed T maze or in a delayed-nonmatching-to-position task (624, 625). Subsequent hippocampal EEG recordings show loss of delta power in galanin-treated rats (624).

Studies further suggest that galanin acts anxiolytically and produces deficits in cued fear conditioning and retrieval of fear memory (626–629).

Despite a wealth of literature, the results described here do not allow clear conclusions on galanin’s role in learning and memory. Likewise, studies on galanin in the context of anhedonia or despair report mixed outcomes in the forced swim and tail suspension test (630–641). The observed differences are likely a reflection of brain region-specific and cell-type specific actions of galanin in the CNS as described in galanin-dependent differences in impulse control between the PFC and ventral hippocampus (559, 642) emphasising the need for brain-region- and/or cell-type-specific manipulations of the galaninergic system (638).

## Oxytocin

### Discovery and expression in the brain

Oxytocin (OXT) was first discovered at the end of the 19^th^ century/beginning of the 20^th^ century showing that i.v. administration of posterior pituitary extracts caused elevations of blood pressure and uterine contractions in different animal species. OXT is a member of the evolutionary conserved oxytocin-vasopressin (OXT-VP) peptide family. Both peptides have emerged from a common OXT-VP-like peptide that first appeared around 600 million years ago (643). Roughly 50 years after the initial descriptions of its physiological effects by Livermore and du Vigneaud, OXT was isolated and its amino acid sequence determined (644, 645). Both peptides consist of 9 amino acids. In the brain, OXT and VP exert their effects via activation of OXTR and AVPR1A, with OXT having a higher affinity for OXTR.

Expression of OXTRs occurs throughout the human and rodent brain (2, 4, 59, 646–648) with slightly higher levels in limbic and olfactory brain areas. Most OXTRs are found on inhibitory INs many of which co-express CCK or SOM (59, 649). In the PFC, the expression of OXTR and basic electrophysiological properties of SOM-OXTR-INs in male and female mice are similar, but SOM-OXTR-INs of female mice may be more sensitive to OXT compared to SOM-OXTR-INs of male mice (59).

### OXT release

OXT and VP are mainly synthesised by projection neurons of the SON, and paraventricular nucleus of the hypothalamus. The processes of OXTergic and VPergic neurons extend to the posterior pituitary gland to enable systemic circulation of both neuropeptides (159, 650–652). Processes of OXTergic/VPergic cells reach the posterior pituitary, but also virtually all brain areas including their home nuclei and the PFC, septum, CeA, BNST, nucleus raphe and LC (643, 647, 648, 653–661).

Basal OXT release underlies a circadian rhythm (132, 133). This basal secretion is increased in response to hormonal changes associated with pregnancy and/or lactation but also in response to sensory stimuli such as suckling or infant cries/pup calls (152–156). In addition, sexual stimulation as well as changes in osmolality and other stressors lead to release of OXT (135–144). OXT is therefore considered an integral part of the stress response: Indeed, OXT is partially colocalised with corticotropin-releasing hormone (CRH) in parvocellular neurons and OXT release is dependent on stress-induced hypothalamus-pituitary-adrenal (HPA) axis activation (144, 662, 663). At the same time, OXT has been shown to attenuate HPA axis function (664, 665).

In the SON, dendritic OXT release was shown by electron microscopy and later confirmed by microdialysis (155, 666, 667). Magnocellular neurons of the SON and paraventricular hypothalamic nucleus release OXT preferentially into the blood, however, both neurons also project differentially to other brain areas: Specifically, paraventricular neurons modulate the activity of neuronal circuits for state control [attention (e.g. LC, pons), threat/defence (e.g. PAG, amygdala), sleep (e.g. PON, SON)], for somatic visceral control [sensory motor regulation (e.g. substantia nigra, globus pallidus), pain (e.g. raphe nucleus, parabrachial nucleus), metabolism (e.g. solitary tract nucleus, dorsal motor nucleus of vagal nerve)], or for cognitive control [reward (e.g. PFC, VTA), learning/memory (e.g. CA2, tuberomammillary nucleus), reproduction (e.g. PON)]. OXTergic projections from SON neurons to these neuronal circuits are much weaker and more restricted (648, 655, 668).

### Cellular and network actions of OXT

OXT inhibits glutamatergic drive onto magnocellular SON neurons via a Ca^2+^-dependent presynaptic inhibition of glutamate release (669). Similarly, OXT induces an inward current leading to membrane potential depolarisation and increase in spontaneous and/or evoked firing in OXT–responsive cells of different brain areas (59, 660, 661, 670–679). In the BNST, PFC, hippocampus and amygdala and possibly all other brain areas, these OXT-responsive cells are – to varying degrees – inhibitory INs, resulting in OXT-mediated increases in GABAergic transmission onto glutamatergic projection neurons (673, 680–683). Further, the allosteric enhancer of GABA_A_Rs diazepam facilitates OXT-mediated inhibitory effects on spontaneous spiking activity in projection neurons of the CeA (684) which is why it has been suggested as a potential add-on therapy in anxiety-related disorders (647). In serotonergic neurons of the nucleus raphe, OXT has been shown to bidirectionally modulate glutamatergic transmission causing sustained potentiations of evoked EPSCs on the one hand and sustained depressions of evoked EPSCs on the other (660). Both mechanisms rely on an OXTR-dependent regulation of presynaptic glutamate release and retrograde endocannabinoid and arachidonic acid signalling. Interestingly, OXT-dependent potentiations and depressions of glutmatergic transmission are cell-type specific such that OXT bidirectionally gates excitatory transmission onto neurons projecting to the mPFC or hippocampus whereas it inhibits glutamatergic transmission onto dorsal raphe neurons projecting to the lateral habenula or CeA (685–688).

In the BLA, activation of GABA_B_ receptors is responsible for the inhibition of glutamatergic drive and synaptic plasticity (681). In A1, activation of a disinhibitory circuit was observed in response to OXT treatment. This disinhibition reduced pup call-evoked inhibitory postsynaptic currents in the left A1 of inexperienced female mice within seconds whereas glutamatergic transmission was only increased within minutes. Altogether, it is suggested that pairing of pup calls with OXTR activation is the basis for changes in maternal behaviour in experienced mice (689).

At the cellular level, it could be shown that OXT-induced facilitation of LTP induction at the CA1-subiculum synapse relies on TRPV1 channel activation (678). In addition, OXT promotes LTP via activation of the PLC signalling pathway in the hippocampal CA1 region of nulliparous parent rats (690), whereas antagonism of OXTR signalling prevents LTP induction in multiparous female mice and impedes spatial maze task performance (691). In addition, intranasal OXT administration after experiencing uncontrollable stress has been shown to rescue LTP induction and to attenuate deficits in object recognition in stressed rats (692).

Further studies suggest that OXT has the ability to modulate synaptic plasticity in the PFC such that high-fat diet-induced impairments in LTP are rescued by microinjection of OXT and that OXT impairs the maintenance of NMDAR-independent LTP (688, 693).

Collectively, these data show that OXT is able to alter learning-dependent behaviours by gating excitation and inhibition in a circuit- and stimulus-dependent fashion.

### OXT-mediated behaviours

It is beyond the scope of the review to summarise all behavioural outputs that are modulated by OXT. Instead, we will focus here on the role of OXT in stress-, anxiety-, and depression-related behaviours, well aware of the fact, that we are only able to offer a snippet of all OXT actions on these behaviours.

### OXT and symptoms of despair

OXT plays an important role in animal survival as it strengthens bonding between parent and offspring. As such, the actions of OXT in rodents and humans are surprisingly similar.

In rodents, OXT administration to the mPFC (694), the paraventricular nucleus of the hypothalamus (695, 696), the CeA (697), the brain ventricles (698) or the periphery (699) results in anxiolytic behaviour in the EPM task. Using an optogenetic approach, OXTR-INs of the mPFC have been identified as key regulators of anxiolysis as they antagonise CRH and block the CRH-induced potentiation of LII/III PC activity (668). In rats, mPFC-dependent anxiolytic OXT effects are accompanied by increased fear resilience investigated in the intruder paradigm (700) and microinjection of the OXTR antagonist atosiban block this resilience. In addition, the OXT-induced anxiolytic effect relies on above-mentioned increased OXT-dependent GABAergic drive as GABA_A_R inhibition prior to OXT microinjection into the mPFC or paraventricular nucleus of the hypothalamus attenuates the OXT-mediated anxiolytic effect assessed in the EPM test (694, 695).

In human PTSD patients, an intranasal dose of OXT decreases subjective levels of anxiety and nervousness (701). In addition, intranasal OXT application enhances Pavlovian fear responses in healthy male individuals and increases BOLD PFC activity in OXT-treated, fear-conditioned compared to non-fear-conditioned subjects and a general inhibition of amygdalar activity (702). In addition, the actions of OXT in both species are thought to depend on an individual’s emotional state. This is exemplified by the finding that OXT in anxiously-attached human individuals triggers rather negative effects compared to less anxiously-attached individuals (703). Further, intranasal OXT application decreases brain activity (measured by the BOLD signal and functional brain imaging) in the amygdala, the PFC including the ACC as well as the insular cortex after trauma-script-driven imagery. These neural effects are accompanied by lower levels of sleepiness and higher flashback intensity (704). Similarly, in human mothers suffering from post-partum depression, intranasal OXT administration is associated with adverse effects towards the baby by enhancing a depressed mother’s negative cognitive processes in response to auditory stimuli from the offspring (705, 706). Conversely, intranasal OXT administration correlates positively with reward circuit activation and positive emotional states (707). These findings raise the question as to whether early life experiences shape responsiveness to OXT. Indeed, low level of maternal care lead to reduced OXTR expression in the, *inter alia*, paraventricular nucleus of the hypothalamus, amygdala and septum of rats (708). Lower levels of OXTR in turn, are associated with less maternal responsiveness towards the offspring (708–710).

In addition, rodent studies on despair and anhedonia suggest that OXT administration attenuates depressive-like behaviours in the forced swim (711) or tail suspension test (712, 713). Disruptions of OXTergic signalling may occur in patients suffering from MDD; however, no clear consensus on OXT levels and MDD is identifiable (714–717). Reasons for the variability in outcome may be the multi-factoriality of the disease leading to a high heterogeneity of patients included in the analysis, making direct comparisons within a patient cohort and across different patient cohorts sometimes difficult.

### OXT and emotional state discrimination

In animals, the ability to distinguish familiar conspecifics and offspring from intruders is critically important to sociability and social behaviour is partly shaped by the processing of chemical signals by the olfactory system. In rats, OXT increases the excitability of PC-like anterior olfactory nucleus neurons and boosts glutamatergic transmission between the anterior and main olfactory bulb (718) resulting in enhanced processing of socially relevant olfactory information. The social interest in an unfamiliar juvenile conspecific is thereby increased and memory of familiar conspecifics is prolonged. Further evidence for a modulation of olfactory processing by OXT is provided by showing that OXT promotes the strength of synaptic transmission in the olfactory tract between mitral to granule cells of the anterior olfactory bulb in an NMDAR-dependent fashion (719).

In addition, OXT has been shown to induce LTD of inputs from the accessory but not from the main olfactory bulb to the MeA in anaesthetised rats. Induction of LTD in MeA in turn is necessary for long-term social recognition memory as measured by exploration time of a novel versus familiar juvenile conspecific (720).

Similarly, OXT modulates visual processing such that it favours bottom-up sensory processing and weakens top-down inputs thereby improving early attentional selection and modulating the salience of external stimuli in humans (721, 722).

Projections of OXTR-expressing glutamatergic neurons of the mPFC to mesolimbic nuclei including the BNST and the BLA further help to encode social memory (679, 723). Likewise, OXTergic projections from the paraventricular nucleus of the hypothalamus to the CeA critically influence a mouse’s ability to discriminate emotional states (neutral, fear, relief) in conspecifics (724). Emotional state discrimination was also shown to be significantly improved in autistic individuals after receiving a single intranasal dose of OXT (725).

Similarly, intranasal OXT administration to healthy male and female individuals significantly increases ratings of empathic embarrassment when confronted with embarrassing situations to others or to self along with a decrease in skin conductance response and decreases in amgydalar and insular activity (726).

In addition, OXT may reduce self-centered behaviour on the basis that it blunts normal bias towards remembering self-attributes and reduces the activity of cortical networks involved in self-processing in healthy male individuals (727).

These data collectively show that OXT influences social recognition and emotional state discrimination both of which may be impaired in neuropsychiatric diseases. However, the data also show that OXT may enhance negative and positive experiences and memories in individuals calling for a context-specific administration of the substance.

## Clinical Translation of Neuropeptide Research

The PFC is crucial for goal-directed behaviour that may be influenced by salience, context and past experiences of a given stimulus. Neurons within the human PFC enable this executive function by signalling conflict, error or past performance and changes of PFC activity may be associated with mental disorders such as MDD. Interestingly, neuropeptide and neuropeptide receptor gene expression is distinctly higher in the human PFC compared to other brain areas, begging the question as to whether neuropeptides play a specific role in the PFC and contribute to goal-directed behaviour. Numerous studies have shown that neuropeptides provide sustained modulations of neuronal activity and synaptic transmission and there is further evidence that exogenously applied neuropeptides may modulate animal and human behaviour. Hypothalamic dysfunctions and/or tumours may be associated with severe symptoms such as dysbalances of plasma sodium/potassium/glucose levels, sleep disturbances, obesity or gigantism underlining the importance of hypothalamic hormones and neuropeptides on human and animal physiology. However, we still lack understanding of neuropeptide actions on prefronto-cortical neurons and PFC-dependent behaviours, partly because of our ignorance of stimulus-specific neuropeptide release within the PFC.

In light of the data provided here and elsewhere, the translation of neuropeptide research into clinical significance is a complex but important task with many unknown variables.

One of the many unknown variables is that of reduced responsiveness following prolonged neuropeptide exposure as a consequence of neuropeptide receptor desensitisation (728–730). In addition, desensitisation also occurs on the level of target molecules that are activated by neuropeptide receptor-dependent signalling cascades (731). In addition, many neuropeptidergic systems display sex-dependent differences in expression levels of neuropeptides or neuropeptide receptors, neuropeptide responsiveness and/or neuropeptide-neuropeptide interactions (732–736). Another unresolved question is that of interaction of different neuropeptide systems that may influence each other’s release or cellular signalling response. Drug specificity is also of crucial importance to drug development and remains a challenge to any synthetic ligand to a natural receptor (737, 738). In addition, GPCRs can from homo- or heterooligomers that may lead to unwanted off-target effects (739). The bioavailability of centrally acting neuropeptide drugs represents another hurdle in the development of effective drugs. The development of so-called small molecules was motivated by developing neuropeptide receptor-binding substances that are able to penetrate the blood-brain barrier and overcome limitations of central bio-availability. However, small molecules and/or blood-brain-barrier penetrating ligands for neuropeptide receptors may lead to increased liver toxicity (740). Despite these challenges, some drugs, e.g. above-mentioned DORAs, have successfully transitioned from basic and preclinical studies to clinical use. Another example is the development of calcitonin-gene related peptide antagonists in chronic migraine patients (741).

## Conclusion

This review aimed at providing information of neuropeptidergic signalling in the mPFC and its potential relevance to neuropsychiatric disorders. Different neuropeptides have been shown to exert robust effects on cortical processing and on animal behaviour, however, an unresolved problem to many studies is the question of endogenous, context-dependent peptide release. The development of aptamer-based sensors that allow the specific adsorption of NPY by optical or electrochemical parameters offers exciting new avenues in the registration of neuropeptide release within a given brain region and may help to decipher time and context of neuropeptide release (742–744). In addition, the development of genetically encoded GPCR sensors and nanobody-enabled monitoring of GPCR states will help to distinguish between neurotransmitter and neuropeptide actions (745, 746) and accomplish our knowledge on neuropeptide release on the one and GPCR signalling on the other hand. RNAscope/proteomic data will further add details to the neuropeptidergic landscape of the brain and, together with optogenetic/chemogenetic tools identify neuropeptidergic pathways that may be activated in response to specific stimuli. Electrophysiological and imaging approaches will further help to determine the cellular and network actions of neuropeptides in a sub-second timescale. Together, these data will broaden our understanding on how neuropeptides modulate mPFC-dependent behavioural outputs and how their (dys-)function might be relevant to mental health.
